# Novel role of mortalin in attenuating HIV-1 Tat-mediated astrogliosis

**DOI:** 10.1186/s12974-020-01912-3

**Published:** 2020-09-20

**Authors:** Priyanka, Renu Wadhwa, Rituparna Chaudhuri, Tapas Chandra Nag, Pankaj Seth

**Affiliations:** 1 Department of Cellular and Molecular Neuroscience, National Brain Research Centre, NH-8, Nainwal Road, Manesar, Gurgaon, Haryana 122052 India; 2 AIST-INDIA DAILAB, DBT-AIST International Center for Translational and Environmental (DAICENTER), National Institute of Advanced Industrial Science & Technology (AIST), Tsukuba, 305-8565 Japan; 3 All India Institute of Medical Sciences, New Delhi, India

**Keywords:** Astrocytes, Glia, HIV-1 Tat, Mortalin, Neuroinflammation, Neuronal survival

## Abstract

**Background:**

In human immunodeficiency virus-1 (HIV-1) infection, activation of astrocytes induces imbalance in physiological functions due to perturbed astrocytic functions that unleashes toxicity on neurons. This leads to inflammatory response finally culminating into neurocognitive dysfunction. In neuroAIDS, HIV-1 protein, transactivator of transcription (Tat) is detected in the cerebrospinal fluid of infected patients. Mortalin, a multifunctional protein, has anti-inflammatory role following its activation in various stress conditions. Recent studies demonstrate downregulation of mortalin in neurodegenerative diseases. Here, we explored the mechanisms of mortalin in modulating HIV-1 Tat-mediated neuroinflammation.

**Methods:**

Expression of mortalin in autopsy section in normal and diseased individuals were examined using immunohistochemistry. To decipher the role of mortalin in HIV-1 Tat-induced activation, human fetal brain-derived astrocytes were transiently transfected with Tat and mortalin using expression vectors. HIV-1 Tat-mediated damage was analyzed using RT-PCR and western blotting. Modulatory role of mortalin was examined by coexpressing it with Tat, followed by examination of mitochondrial morphodynamics using biochemical assay and confocal and electron microscopy. Extracellular ATP release was monitored using luciferase assay. Neuroinflammation in astrocytes was examined using flow cytometry, dye based study, immunocytochemistry, immunoprecipitation, and western blotting. Indirect neuronal damage was also analyzed.

**Results:**

HIV-1 Tat downregulates the expression of mortalin in astrocytes, and this is corroborated with autopsy sections of HIV-1 patients. We found that overexpression of mortalin with Tat reduced inflammation and also rescued astrocytic-mediated neuronal death. Using bioinformatics, we discovered that binding of mortalin with Tat leads to Tat degradation and rescues the cell from neuroinflammation. Blocking of proteosomal pathway rescued the Tat degradation and revealed the ubiquitination of Tat.

**Conclusion:**

Overall, our data demonstrated the protective role of mortalin in combating HIV-1 Tat-mediated damage. We also showed that mortalin could degrade Tat through direct binding with HIV-1 Tat. Overexpression of mortalin in the presence of Tat could significantly reduce cytotoxic effects of Tat in astrocytes. Indirect neuronal death was also found to be rescued. Our in vitro findings were validated as we found attenuated expression of mortalin in the autopsy sections of HIV-1 patients.

## Introduction

Despite the success of combinatorial anti-retroviral therapy (cART), human immunodeficiency virus-1 (HIV-1) remains a major health challenge worldwide as the morbidity in HIV-1 patients and new infections are still reported [1]. In general, HIV potentially infects the immune system and hijacks its machinery for viral replication, thereby weakening the natural defence system and severely affecting the immune mechanism [2]. In addition to systematic circulation, HIV-1 can infect the central nervous system (CNS) by breaching the Blood-Brain Barrier (BBB) [3, 4]. Invasion of HIV in CNS and causing damage to neuronal cells ultimately results in neurocognitive decline, motor impairment, deterioration in speech, and memory loss.

Though cART turns the almost fatal disease to chronic low lying infection, it fails to effectively suppress the production of HIV-1 viral proteins in the brain. The prevalence of HIV-1 associated neurocognitive disorder (HAND) continues to rise due to sustained HIV-1 infection in brain that act as viral reservoirs. HIV-1 Tat is a regulatory protein that is released and efficiently taken up by many cell types including astrocytes [5]. Astrocytes are extensively infected with HIV-1, and these cells also act as a “safe heaven” for the latent form of the virus [6, 7]. In addition, the clinical reports also reveal the presence of HIV-1 Tat protein in the cerebrospinal fluid (CSF) from the infected patients successfully treated with cART [8].

In CNS, there are three major types of cells: microglia, astrocytes, and neurons involved in inducing direct and indirect neuroinflammation [9, 10]. The infection causes dysregulation in brain homeostasis [11] and affects the cellular and molecular machinery of the system. Astrocytes are the most abundant cell type of the brain. They perform various critical functions such as modulating synaptic transmissions, ionic metabolism, provides growth factors to neuron, and in structural integrity of tight junction in BBB [12]. In HIV-1-associated pathological conditions, astrocytes exhibit an aggravated response leading to several structural and functional dysfunctions such as increased inflammation by inducing cytokine and chemokine release, NF-kβ activation [13, 14] prolonged ROS secretion, enhanced mitochondrial fragmentation, and decreased mitochondrial membrane potential [15, 16]. It has been observed that extracellular ATP activates P2X7 receptors followed by the induction of rapid release of virion particles accumulated in virus containing compartments (VCC) from human macrophages [17]. Extracellular ATP burst is highly uncontrolled in HIV-1 infection, which affects the neuronal spine density and neuronal health. Furthermore, other signaling pathways are also dysregulated in astrocytes such as increased calcium (Ca^2+^) influx and reduced glutamate uptake due to altered glutamate receptors [18]. Phospholipids also play an important role in regulating the Tat entry in the cells and the high affinity of Tat with phosphatidylinositol 4,5-bisphosphate strongly affect the Tat secretion in the system [19, 20]. Together, these alterations disrupt the neuro-glia crosstalk and induce neuroinflammation causing direct and indirect neuronal death.

The integrated HIV DNA has been detected in astrocytes in post-mortem tissue of the HIV-1-infected patients [21]. The aggravated response of HIV-1 to astrocytes leads to astrogliosis with remarkable alteration in molecular and functional activity of the neighboring cells. The amplified astrogliosis spreads the toxic signals and induces direct and indirect neuronal death by various known and unknown mechanisms such as increased inflammatory response, reduction in extracellular glutamate uptake, and increased mitochondrial damage [22, 23].

Heat shock protein (HSP) is highly conserved and involved in routine biological processes such as maintenance of cellular homeostasis, prevention of protein aggregation, and protein misfolding. Among them, a dynamic protein, mortalin, is a member of heat shock 70 (HSP70) family protein. Mortalin has multiple subcellular locations including cytosol, endoplasmic reticulum, plasma membrane, and mitochondria [24]. In mitochondria, mortalin is critical for import and export of various proteins, maintains mitochondrial biogenesis, and helps in ATP generation [25, 26].Beside that, mortalin is also known for its multiple binding partners localized in various parts of the cell. It regulates cell survival [27] in stressed conditions such as oxidative stress [28], ischemic conditions, and glucose deprivation that causes increased expression of mortalin as an adaptive response, whereas cancer cells possess enriched levels of mortalin [29]. Interestingly, expression of mortalin in Alzheimer’s and Parkinson’s disease are downregulated both in clinical samples and culture cells [30, 31]. In addition, the mitochondrial localization of mortalin helps in regulating the mitochondrial health. Knockdown of mortalin exhibits marked reduction in neuronal mitochondria in synapses [32]. In HIV-1 and other neurodegenerative diseases, alleviated pathology to mitochondria in culture cells which was substantiated with clinical studies [16, 33].

Modulation of mortalin is extensively studied in mild form of stress [27, 28] and cancer cell [34], whereas the understanding of the mortalin remains enigmatic in virus-induced neurodegeneration. As there are no studies on the role of mortalin in HIV-1-mediated neuropathogenesis, we initiated the study to investigate the modulation of mortalin levels in Tat-transfected astrocytes and its indirect effect on neuronal health. We also checked the mortalin levels in autopsy brain sections of HIV-1 patients and levels of mortalin in human astrocytes following overexpression of HIV-1 Tat. We performed an in-depth study to understand the correlation of mortalin and HIV-1 Tat in human fetal brain-derived astrocytes.

## Material and methods

### Cell culture

#### Human fetal brain cell culture

Aborted human fetal brain tissue from 10–15 week gestation was collected after informed consent from mother. The tissue was processed under the strict guidelines of the institutional Human Ethics Committee and Stem Cell Research Committee of National Brain Research Centre (NBRC), India, in compliance with the recommendation of Department of Biotechnology (DBT) and Indian Council of Medical Research (ICMR), India. Human neural progenitors cells (hNPCs) were isolated from the SVZ region, and cells were seeded on the poly-d-lysine (PDL) (Sigma–Aldrich, St. Louis, MO) containing neurobasal media (Invitrogen, San Diego, CA, USA) supplemented with Neural Survival Factor-1 (Lonza, Charles City, IA), N2 supplement (Invitrogen, San Diego, CA, USA), bovine serum albumin (Sigma–Aldrich, St. Louis, MO), glutamine (Sigma, St. Louis, MO), 25 ng mL^−1^ bFGF (Peprotech, Rocky Hill, NJ, USA), and 20 ng mL^−1^EGF (Peprotech, Rocky Hill, NJ, USA). Before any experiments, cells were characterized using neural stem cell markers, and it was found to be 99.9% positive for Nestin and SOX2. At different passages, cells were also differentiated into astrocytes and neuron, followed by their characterization by immunostaining and PCR, almost 99% of astrocytes were positive for glial fibrillary acidic protein (GFAP), and neurons expressed microtubule associated protein-2 (MAP2) respectively, they were also found to be negative for Nestin and SOX2. In this study three different fetuses (age 12 weeks, 14 weeks, and 16 weeks) were used, and results were comparable in all of them.

#### Primary human neural progenitor-derived astrocytes (PDA)

Isolated neural progenitor cells were differentiated into astrocytes and were referred to as progenitor-derived astrocytes (PDA). To initiate the differentiation in NPCs, Neurobasal media was replaced with astrocytic media—comprising with Eagle’s minimum essential medium (MEM) (Sigma–Aldrich, USA) containing 10% heat inactivated fetal bovine serum (FBS) (Gibco, Invitogen) with pen-strep and gentamycin called the complete MEM (CMEM) media. At every alternate day, half media was replaced with fresh CMEM till the maturation. Astrocytes were stained with glial markers to assess the purity using immunocytochemistry and PCR, more than 99% cell were positive with GFAP (Dako, Glostrup, Denmark), S100β, and ALDHDL1 (Santa Cruz Biotechnology, Santa Cruz, CA, USA), and fully differentiated cultured astrocytes were used in subsequent experiments.

#### Primary human neuronal culture

Isolated NPCs were seeded on poly-d-lysine coated flasks with neuronal media containing Neurobasal media, bovine serum albumin (BSA) (Sigma–Aldrich, USA), N2 supplement (Invitrogen, San Diego, CA, USA), and Neural Survival Factor-1 (Lonza, Charles City, IA), supplemented with 10 ng/ml Platelet-derived growth factor (PDGF) (Peprotech, Rocky Hill, NJ, USA) and 10 ng/ml Brain-derived growth factor (BDNF) (Peprotech, Rocky Hill, NJ, USA). Half media was changed till maturation on every alternate day, followed by characterization for neuronal markers, and 99% of the cells were Tuj-1 and Map2 positive.

#### Plasmids and transfections

Full length (101 aa) pcDNA3.1 HIV-1 Tat B and pcDNA3.1 mortalin-V5 expression plasmids were kind gifts from Prof. Udaykumar Ranga, JNCASR, India, and Dr. Renu Wadhwa, AIST, Tsukuba, Japan, respectively. Cells were transfected at the confluency of 75–80% using Lipofectamine 2000 (Invitrogen, San Diego, CA, USA) as per the manufacturer’s protocol. For the transfection, 3 μg of HIV-1 Tat B and mortalin plasmids were used for the transfection in T25 flask, 1.5ug of plasmid for 6-well plate, 700 ng for 12 well plate, 250 ng for 4-well chamber slide, and 100 ng for 8-well chamber slide, and same concentration was used for the plasmid control. In the transfected samples, the PC stands for the plasmid control, and PC + PC is the two plasmid controls for the cotransfection. The backbone of both mortalin and HIV-1 Tat plasmid is the pcDNA3.1. The PDA were transfected for 24 h. Transfected cells were processed for various experiments and assays. For indirect death or astrocytic-mediated neuronal damage, the astrocytic conditioned media was collected from the astrocytes post transfection for 24 h and added to primary cultures of neurons in 1:1 ratio. Neuronal health was assessed after 24 h of treatment. The transfection efficiency of all vectors in PDA was 20–25% (Fig. S.2.) by Lipofectamine 2000 and was comparable.

#### Mutant tat

Wild-type C-Tat contains cysteine and serine residues at positions 30 and 31, respectively (CS); for mutant experiments, site-directed mutation was done in Tat-C—with CC (cysteine-cysteine) and SC (serine-cysteine) at position 30 and 31 (Mishra M et al.). These mutated Tat were used for transfecting the PDA for 24h using Lipofectamine 2000 (Invitrogen, San Diego, CA, USA) as per the manufacturer’s protocol.

#### siRNA-mediated knockdown of mortalin

Mortalin knockdown was achieved by transfecting siRNA of mortalin (Dharmacon, ON-TARGET plus, Human) to downregulate the levels of mortalin in astrocytes. siRNA (100 pmols) was transfected using RNAi Max (Invitrogen, USA) according to manufacturer’s protocol. All Stars Negative control siRNA (Sigma-Aldrich) was used as control.

### Western blot analysis

For western blot analysis, protein was isolated using SDS lysis buffer (0.5 M Tris pH 7.5, NaCl 3 M, NaF 1 M, Na_3_O_4_V 0.5 M, EDTA 0.5 M, SDS 20% and NaB 100 mM and protease inhibitor (Roche, Mannheim, Germany) from transfected PDA followed by sonication). After determining the protein concentration using bicinchoninic acid (Sigma), protein was separated by sodium dodecyl sulfate-polyacrylamide gel electrophoresis (SDS-PAGE) using 12% gel. Separated proteins were transferred on nitrocellulose membrane (MDI, Ambala, India). For immunodetection, the blots were processed for blocking with 5% skimmed milk at room temperature for 2 h, followed by incubation with primary antibodies: anti-HIV-1 Tat(N.B.R.C.), anti-Nf-kB p-65 (1:1000, Cell signalling), anti-phosphorylated-Nf-kβ p-65 (1:1000, Cell signalling), anti-COX IV (1:2000, Cell signalling), anti-GAPDH (1:1000, Santa Cruz, Biotechnology, SantaCruz, CA, USA), and anti-mortalin(1:2000, kind gift from Dr. Renu wadhwa, AIST, Japan) overnight at 4 °C. After washing with 1X TBST thrice for 5 min, blots were incubated with HRP conjugated secondary antibodies (1:2000, Vector Labs, Burlingame, CA, USA) for 1 h at room temperature. After the incubation blots were extensively washed with 1X TBST for five times at the interval of 5 min, the protein detection chemiluminescence reagent (Milipore, Bedford, MA, USA) was used and imaged with ChemiGenius Bio-imaging System (Syngene, Cambridge, UK). Densitometry analysis of developed blots was carried out using the ImageJ software (NIH), and normalization was done using the respective internal control. Representative blots are depicted in figures.

#### Separation of cytoplasmic and nuclear protein

In order to analyze the translocation of protein from cytoplasm to nucleus, transfected samples were isolated using HEPES (cytoplasmic isolation) and 1X SDS buffer (nuclear isolation). Post transfection, cells were first scraped out using HEPES buffer (HEPES buffer pH 7.6, 10 mM MgCl_2_, 100 mM KCl, 100 mM sodium butyrate, 200 mM glycerol phosphate, and Triton X-100 in autoclaved MQ) with protease inhibitor cocktail. Isolated cells were incubated on ice briefly for 30 min with vortexing after every 10 min followed by centrifugation at 12,000 rpm at 4 °C for 30 min. Supernatant was collected in fresh tube and 40ul of 1X SDS buffer comprising of Tris (pH 7.5), 150 mM NaCl, 50 mM NaF, 1 mM EDTA, 1 mM Na_3_VO_,_ 2% SDS, and protease inhibitor cocktail tablet in MQ was added to the pellet, and both the fractions were processed separately. Both the nuclear and cytoplasmic proteins were processed for sonication followed by centrifugation at 12000 rpm for 30 min at 4 °C for cytoplasmic protein and 25 °C for nuclear protein. Protein was estimated using BCA method and processed for immunoblotting.

#### Co-immunoprecipitation assay

To analyze the protein-protein interaction, after 24 h of transfection, cell was lysed using NP-40 lysis buffer (Tris pH 8.6, 10 mM EDTA, 02 mM EGTA, 0.5% NP-40, Na_3_VO_4_, 1% TritonX-100, 150 mM NaCl) containing phosphatase inhibitors (10 mM NaF and 0.1 mM Na_2_VO_5_) and tablet protease inhibitor cocktail. Lysed cells were briefly sonicated and centrifuged for 10,000×*g* at 4 °C for 10 min. Supernatant was collected and processed for immunoprecipitation. For each immunoprecipitation, 500 μg of estimated protein using BCA method was either incubated with 4 μg of mortalin or HIV-1Tat antibodies, while sample was incubated with IgG antibody. After 12 h of incubation at 4 °C, next, pre-cleaned 40 μl G-agarose beads was added to each microfuge tube containing samples at 4 °C on for 16 h. The above complex was washed 5 times with NP-40 buffer to remove the unbound proteins. Proteins bound with beads were eluted into 1X SDS sample buffer and boiled for 7 min. The eluted proteins were further processed for immunoblotting.

#### Quantitative real-time PCR

RNA was extracted from the transfected samples using TRIZOL (Sigma-Aldrich) according to the manufacturer’s protocol. cDNA was synthesized from the RNA using high-capacity cDNA reverse transcription kit (Applied Biosystems, Austin, TX, USA) as per the manufacturer’s instructions. Real-time PCR was performed with SYBER Green master mix (Applied Biosystems) using following primers for mortalin and GAPDH: mortalin forward 5′-CTCGTGGATTCCTCAGATTG-3′ and mortalin reverse 5′-CTCACGTCCTGTGCCTTTAT-3′; GAPDH forward 5′-GAAGGTGAAGGTCGGAGTC-3′ and GAPDH reverse 5′-GAAGATGGTGATGGGATTTC-3′. The cycling conditions used were 95 °C for 3 min (1 cycle), 95 °C for 20 s, 62 °C for 30 s, and 72 °C for 40 s (35 cycles).

### Imaging

#### Immunocytochemistry

For immunocytochemistry (ICC), cells were seeded in 4-well chamber slide (Nunc, Kamstrupvej, Denmark) at the density of 20,000 cells per well. After transfection cells were fixed using 4% paraformaldehyde (PFA) for 20 min followed by washing with 1X PBS, cells were blocked and permeabilized with 4% bovine serum albumin (Sigma-Aldrich, USA) with 0.25% TritonX-100 for 2 h at room temperature. After this, cells were incubated with following primary antibodies for overnight at 4 °C: anti-GFAP (Dako, Glostrup, Denmark; 1:1000), anti-mortalin (kind Gift from Dr. Renu Wadhwa laboratory, AIST, Japan; 1:3000), anti-Nestin (Milipore; 1:1000) anti-Map2 (Chemicon, 1:1000), and anti-HIV-1 Tat (raised in rabbit at National Brain Research Centre, 1:500) followed by washing with 1X PBS thrice, and appropriate secondary antibodies tagged with Alexa Flour 594 and Alexa Flour 488 (1:500;Invitrogen, San Diego, CA, USA) were incubated in dark for 2 h. At the final step, cells were mounted with hard set mounting media containing DAPI (Vector Labs, Burlingame, CA, USA). Minimum 9–10 images were captured from random fields using AxioImager.Z1 microscope (Carl Zeiss, Heidenheim, Germany).

#### Immunohistochemical

For immunohistochemical staining, 5 μm thick sections were obtained from the Human Brain Tissue Repository, National Institute of Mental Health and Neurosciences, Bangalore, India. The non-HIV controls were age matched controls of cases from road trauma accidents with no known neurological disorders. The HIV-1 cases were drug naïve and were not on any antiretroviral therapy. The control non-HIV infected (*n* = 3) and HIV-1 infected without any opportunistic infections (*n* = 3) with mild neurocognitive impairment were processed. For antigen unmasking solution at 70 °C for 40 min followed by washing with PBS, endogenous peroxidases were quenched using quenching solution (10% H_2_O_2_, 10% methanol in 1X PBS) for 15 min and washed with 1X PBS. Next, sections were permeabilized using 0.3% Triton X-100 for 10 min and blocked with blocking solution (3% normal goat serum, 1% bovine serum albumin, and 0.3% Triton X-100) for 2 h at room temperature. Sections were incubated with mortalin antibody at the dilution of 1:250 briefly for 48 h at 4 °C. After incubation, sections were washed with PBS and incubated with biotinylated secondary antibody at a dilution of 1:500 for 2 h, and sections were washed and further incubated with VECTASTAIN-Elite ABC solution for 2 h as per the manufacturer’s protocol. Stain was developed using ImmPACT Novared peroxidase substrate kit. Minimum 10 images were captured randomly using bright field from Leica DM RXA2.

#### Mitochondrial morphology

PDA were seeded on cover glass at the density of 20,000 cell/well, followed by transfection with HIV-1Tat, mortalin, cotransfection of mortalin and HIV-1 Tat, and empty vectors (denoted as PC—plasmid control). After 24 h of transfection, full media was replaced with fresh media with MitotrackerRED CMXRos (Invitrogen, San Diego, CA, USA) and incubated at 37 °C for 10 min in dark. Next, cells were immediately washed with 1X PBS followed by quick cell fixation using 4% paraformaldehyde (PFA) for 20 min in dark at room temperature. Next, cells were processed for blocking (4% BSA with 0.25% Triton X-100 in PBS) for 1 h at room temperature. Coverslips were mounted with hard set mounting medium DAPI. We only analyzed the clearly visible mitochondria and avoided the dense and highly entangled one. Minimum 20 images were captured randomly using Eclipse Ti2 inverted microscope at × 60.

#### Electron microscopy

PDAs were seeded and transfected with HIV-1Tat, mortalin, and empty vector for 24 h. Cells were isolated using trypsin and pelted down followed by washing with culture grade PBS. Next, cells were fixed using EM grade glutaraldehyde fixative buffer for 1 h at 4 °C. After incubation, fixative was washed out with PBS, and cells were post fixed in 1% psmium tetroxide for 30 min. In order to dehydrate the cells, ascending grade of acetone was used followed by infiltration and embedding in araldite CY 212 (TAAB, UK). Thin sections (60–70 nm) were cut and further stained with uranyl acetate and alkaline lead citrate briefly for 1 min. Images were captured under Philips CM10 transmission electron microscope at an operative voltage of 80KV. Minimum 5 images were randomly captured from single sample.

#### Indirect neuronal damage assay

The neurons were seeded on glass bottom 4-well chamber slide at the 40,000 cells/well. Post transfection in PDA, the astrocytic condition media was collected and supplemented on the neuron for next 24 h. After that, neurons were washed with 1X PBS and fixed with 4% PFA for 20 min followed by three washing with 1X PBS and blocked using 4% BSA with 0.15% TritonX-100 in PBS. The neurons were labeled with primary antibody-anti-Map2 (1:1500) and incubated overnight at 4 °C; next day, three washed were given to the neuron with 1XPBS at the interval of 5 min followed by secondary incubation Alexa flour 594 (1:500;Invitrogen, San Diego, CA, USA) in dark for 2 h. Next, secondary washes were given four times at the interval of 5 min, and cells were fixed with hardset mounting media DAPI (Vector Labs, Burlingame, CA, USA). Random 10 images were taken from Eclipse Ti2 inverted microscope at 60X.

### Biochemical assays

#### Mitochondrial membrane potential assay

The mitochondrial membrane potential (MMP) was assessed in all transfected cells after 24 h. MMP was measured using JC-1 dye (5,5′,6,6′-tetrachloro-1,1′,3,3′-tetraethyl benzimidazolyl-carbocyanine iodide) (Invitrogen, San Diego, CA, USA), a lipophilic, cationic dye that exhibits a fluorescence emission shift upon aggregation from 530 (green “J-monomer”) to 590 nm (red “J-aggregates”) at the concentration of 1 μg/ml for 15 min at 37 °C. After quick wash with 1X PBS, cells were harvested using cold PBS, and cells were centrifuged at 350*g* followed by a wash with 1X PBS and read at 590 nm/530 nm using Tecan Infinite® 200 PRO.

### Measurement of intracellular ROS

The production of intracellular ROS was monitored by DCFDA fluorescent dye in transfected PDAs, and cells were seeded at the density of 2 × 10^4^ cells per well in a 24-well plate. After 24 h of transfection, full media was replaced with fresh media containing DCFDA dye (Sigma-Aldrich, USA) and incubated for 30 min at 37 °C. Next, cells were washed with 1X PBS followed by cell isolation using NP-40 lyses buffer and immediately measured at 480 nm and 510 nm using Tecan infinite PRO 200.

### The adenosine triphosphate (ATP) release assay

Astrocytes were cultured in a 4-well chamber slide at a density of 20,000 cells/well. After 24 h transfection, full media was replaced with fresh CMEM, followed by collection of supernatant at 1, 5, 10, 15, and 30 min. Extracellular ATP release was measured in supernatant using an ATP bioluminescent assay kit (sigma Aldrich, St. Louis, MO) as per the manufacturer’s protocol. Luminescence was measured by mixing 100 μl assay mix with 10 μl of the sample using single-tube Sirius luminometer (Berthold detection systems, Pforzheim, Germany).

### Assessment of indirect adenosine triphosphate (ATP) release

To assess ATP release in neurons, astrocytes and neurons were cultured. Cultured astrocytes were transfected with HIV-1 Tat, mortalin, and empty vector for 24 h. Post transfection, astrocytic conditioned media was supplemented on neurons for 24 h. Post treatment, full neuronal media was replaced with fresh media followed by collection of supernatant at 1, 5, 10, 15, and 30 min. Extracellular ATP release was measured in supernatant using an ATP bioluminescent assay kit (Sigma Aldrich, St. Louis, MO, USA) as per the manufacturer’s protocol. Luminescence was measured by mixing 100 μl assay mix with 10 μl of the sample using single-tube Sirius luminometer (Berthold detection systems, Pforzheim, Germany).

### Extracellular glutamate release

Astrocytes were cultured on a 24-well plate at the density of 20,000 cells/well and transfected briefly for 24 h with HIV-1 Tat, overexpressed with mortalin, cotransfection of mortalin with HIV-1 Tat, and empty vector. Supernatant was collected and processed for the detection of glutamate release using Glutamate kit (Glutamine and glutamate determination kit, Sigma-Aldrich, USA) as per the manufacturer’s protocol.

### Cytokine bead array

Astrocytes were cultured on a 24-well plate and on the next day, cells were transfected with HIV-1 Tat, overexpressed with mortalin and cotransfection of mortalin with HIV-1 Tat and their respective empty vector (control) for 24 h, followed by the collection of supernatant. To determine cytokine and chemokine levels, supernatant was incubated with cytokine bead (CBA; Multiplex magnetic bead-based antibody detection kits, BD Biosciences, CA, USA) as per the manufacturer’s protocol. Incubated complex was passed by flow cytometer, and represented data was analyzed using the BD FACSDiva software.

### Death assay

#### Determination of neuronal death

Astrocytes were seeded at a density of 20,000 cell/well in a 4-well chamber slide, and cells were transfected with HIV-1 Tat, overexpressed with mortalin, cotransfected with mortalin and HIV-1 Tat, and with their respective empty vector (control) briefly for 24 h, and this half astrocytic conditioned media (ACM) was collected and supplemented on neurons to assess the indirect neuronal death. To assess the indirect neuronal death, treated neurons after 24 h were fixed with 4% paraformaldehyde (PFA) for 20 min. after fixation cell were blocked and permeabilized with 4% bovine serum albumin with 0.25% Triton-X-100 for 30 min, terminal deoxynucleotidyl transferase biotin-dUTP nick end labelling (TUNEL) assay was performed to analyze the neuronal death using the in situ cell Death Detection Kit, TMR red (Roche, Mannheim, Germany) as per the manufacturer’s protocol, and nuclei were stained with DAPI. Images from at least 10 random fields were acquired using AxioImager.Z1 microscope (Carl Zeiss, Heidenheim, Germany). More than 200 cells were counted from each transfected group. Apoptotic neurons were expressed in fold change of TUNEL +ve neurons/DAPI.

## Results

### Mortalin is expressed in human brain autopsy sections and human fetal brain-derived astrocytes

**Fig. 1:**
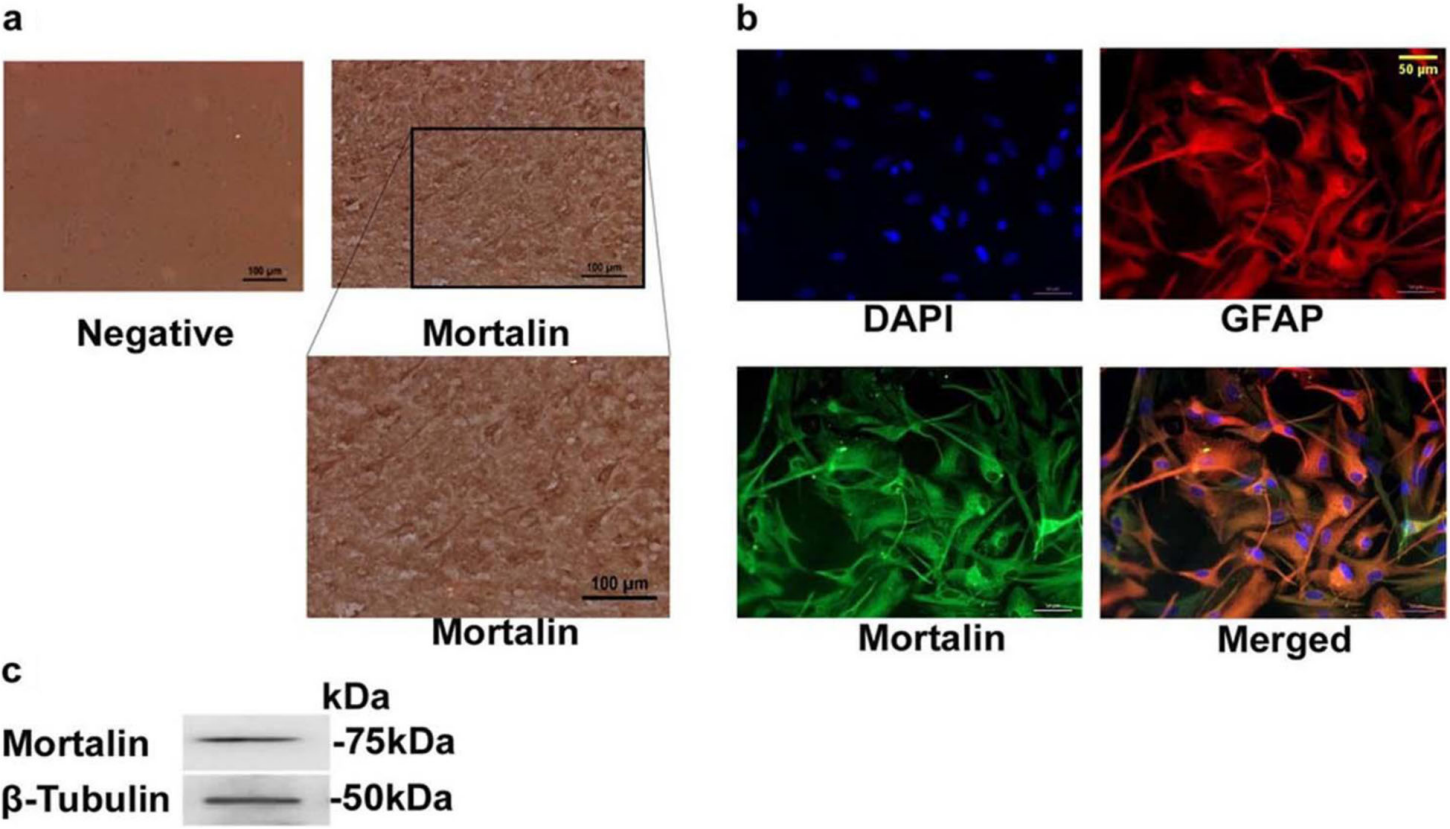
Expression of mortalin in human brain autopsy sections and human fetal neural progenitor derived astroctyes. **a**) Representative immunohistochemical images of human frontal cortex autopsy section showing mortalin positive cells (brown) (**a**, upper right panel). Lower panel represents its magnificent view. DAB stained sections with no primary antibody control shown in left panel (negative). Scale bar, 100 μm (*n* = 3). **b**) Representative immunofluorescence images of PDA co-stained with anti-mortalin in green and anti-glial fibrillary acidic protein (GFAP) in red showing and merged depicting co-localization of mortalin and GFAP. Scale bar, 50 μm (*n* = 4). **c**) Representative immunoblots of total cell lysate of PDA probed with indicated antibodies showing presence of mortalin

We first assessed the expression of mortalin in human frontal cortex tissue obtained from post-mortem autopsy of non-HIV-1 individuals. Immunohistochemistry of these sections showed abundant localization of mortalin in human brain cells (Fig. 1a). To examine the role of mortalin in the human astrocytes in HIV-1 Tat condition, we used well characterized human astrocytes that were differentiated from human fetal brain derived neural progenitor cells using protocols previously established in our lab [35, 36]. We first determined the localization of mortalin in progenitor derived astrocytes (PDA). Immunofluorescence of anti-mortalin stained PDA showed purely cytoplasmic expression of mortalin (Fig. 1b). Expression of mortalin was also analyzed in the human neuronal progenitor and neuronal cells (Fig. S1). Expression of mortalin at protein level was also confirmed in whole cell lysate of PDA by western blotting (Fig. 1c).

### HIV-1 Tat downregulates the expression of mortalin in PDA

**Fig. 2:**
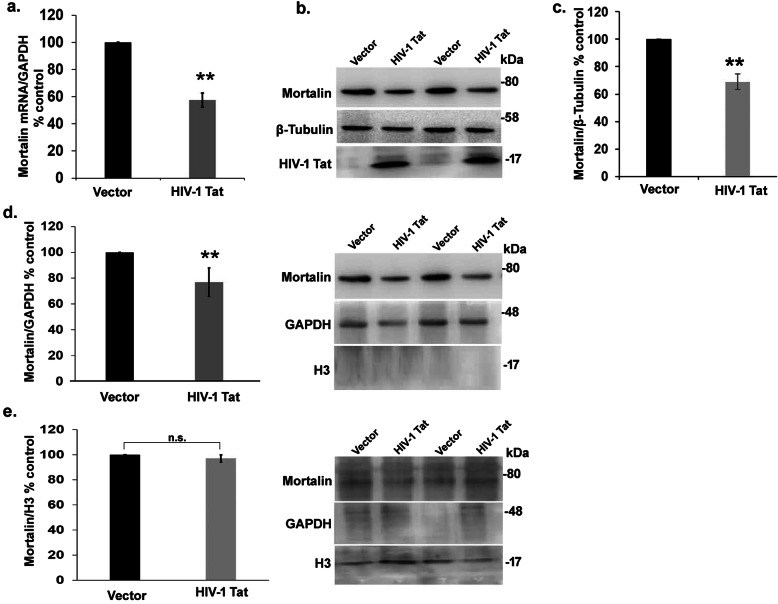
Tat reduces the expression of mortalin without altering its localization. **a**) percentage change in mRNA expression of mortalin in HIV-1 Tat-transfected astrocytes as analyzed by RT-PCR. GAPDH was used as the internal control (*n* = 5). **b**) Representative western blot showing decrease in mortalin level in HIV-1 Tat transfected astrocytes (*n*=5). **c**) Percentage change in mortalin level in HIV-1 Tat-transfected astrocytes as analzed by western blot. β-tubulin was used as the loading control. **d**) Bar graphs represent the percentage change in mortalin level in cytoplasmic extract of HIV-1 Tat transfected astrocytes. (Right panel) Representative western blot showing decrease in mortalin level in HIV-1 Tat transfected astrocytes in the cytoplasmic extract. **e**) Bar graphs represent the percentage change in mortalin level in nuclear extract of HIV-1 Tat transfected astrocytes. (Right panel) Representative western blot showing no change in mortalin level in HIV-1 Tat transfected astrocytes (*n* =3 ). Total histone3 (H3) was used as nuclear control, and GAPDH was used as cytoplasmic control. All data represent mean ± standard deviation, from independent experiments, **p< 0.005 (n.s. stands for non-significant. *n* indicates the number of independent experiments)

Under mild stress conditions such as oxidative stress, ischemic injury, and glucose deprivation [25, 27], mortalin plays a pivotal role to combat the stressful condition by inducing its own expression. High levels of mortalin, on the other hand, contribute to the survival of cancer cells such as breast adenocarcinoma and lung and ovarian cancer [29, 37, 38]. The current study was initiated to understand the role of mortalin in HIV-1 neuropathogenesis. To determine the effect of HIV-1 Tat on mortalin, PDA were transfected using HIV-1 Tat expression vectors, and levels of mortalin were assessed at both mRNA and protein level. RT-PCR assays revealed that mortalin was reduced to almost half in HIV-1 Tat-transfected PDA as compared to control cells (Fig. 2a). This was corroborated by Western blot assay which revealed decreased levels of mortalin (approximately 40%) after 24h (Fig. 2 b, c). There are studies that suggest nuclear shuttling of mortalin promotes cell proliferation and contributes to cell survival [39]. However, this translocation is only reported in cancerous condition till date, hence to investigate whether HIV-1 Tat affects nucleocytoplasmic translocation of mortalin, we fractionated HIV-1 Tat transfected cell into cytoplasmic and nuclear fractions and expression level of mortalin was analyzed by Western blotting. Significant downregulation of mortalin was seen in the cytoplasmic fraction (Fig. 2d), but no change was observed in the nuclear fraction (Fig. 2e). This suggested that HIV-1 Tat downregulated cytoplasmic mortalin expression in PDA, without affecting its translocation.

### Overexpression of mortalin reduces the expression of HIV-1 Tat in PDA

**Fig. 3:**
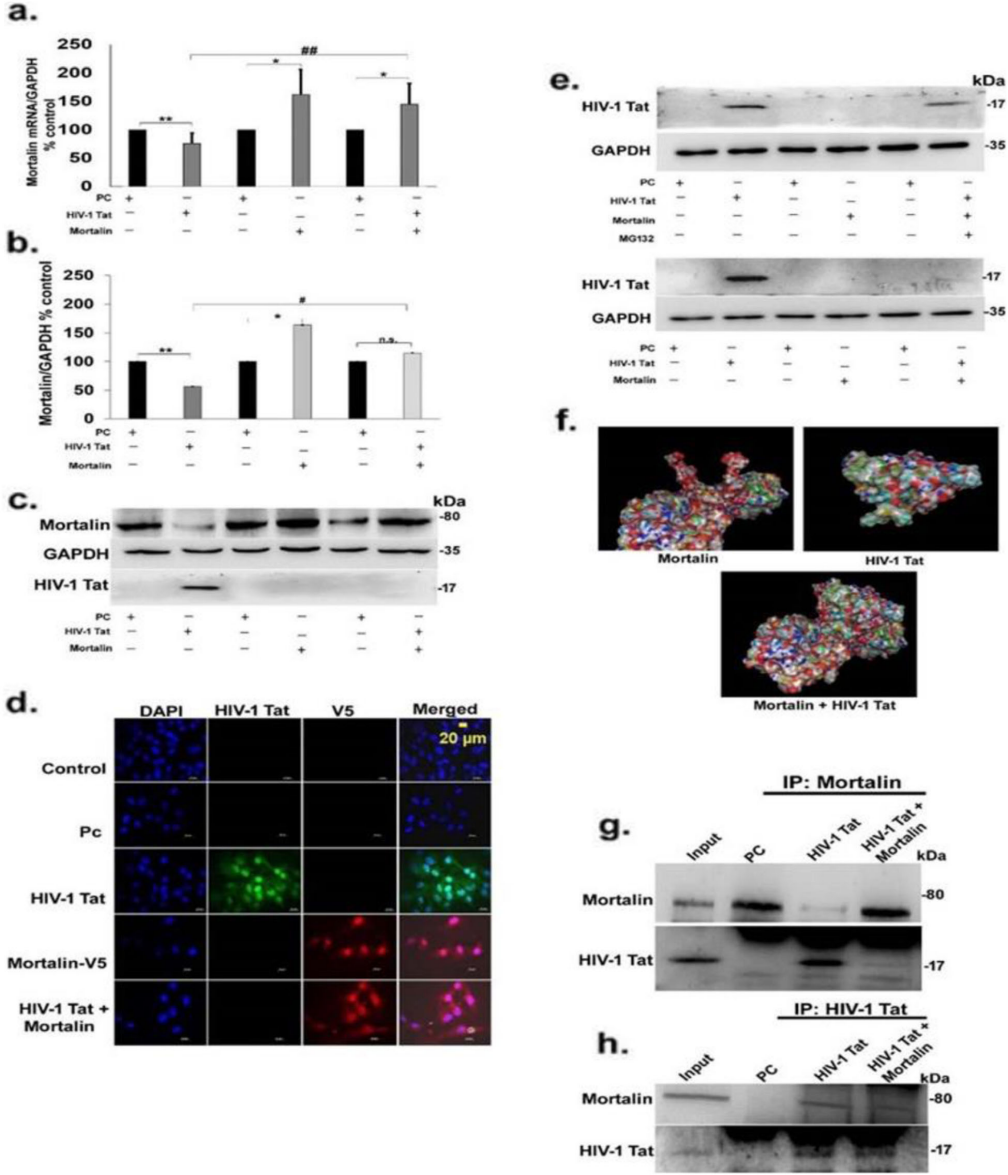
Overexpression of mortalin promotes HIV-1 Tat degradation by ubiquitination. **a**) Percentage change in mRNA expression of mortalin in indicated trasnfected group analyzed by RT-PCR, GAPDH was used as internal control (*n* = 5). **b**) Percentage change in the expression of mortalin at protein level in indicated transfected groups, **c**) analyzed by western blot, GAPDH was taken as loading control (*n* = 5). **d**) Representative immunofluorescence image showed the expression of exogenous mortalin tagged with V5 and HIV-1 Tat in all transfected PDA after 24 h of transfection, scale bar 20 μm (*n* = 3). **e**) Representative western blot showed the expression of HIV-1 Tat protein in transfected cell upon treatment with MG132, indicated by “+” and “-” represents presence and absence of MG132 (*n*=3). GAPDH was used as loading control, and lower panel of western blot represents the expression of HIV-1 Tat in trasnfected cell without MG132 (*n* = 4). **f**) Pictorial representation of docked molecule of HIV-1 Tat and mortalin of substrate-binding site. **g**, **h**) Immunoblots of coimmuniprecipitated PDA of input (control). PC (plasmid control), HIV-1 Tat, and cotransfected (mortalin and HIV-1 Tat) protein probed with anti-mortalin and anti-Tat antibodies, **h**) Represent the reverse CO-IP (*n* = 4). All data represent mean ± standard deviation, from independent experiments (*n* stands for number of independent experiments), *p< 0.05, **p< 0.005 with respect to control, #*p*< 0.05, #*p*< 0.005 with respect to HIV-1 Tat and contransfected group (PC stands for pasmid control and PC+PC is used as plasmid control in cotransfected cells)

In order to uncover the role of mortalin under HIV-1 Tat expressing conditions, we coexpressed mortalin and HIV-1 Tat in PDA using expression vector. Mortalin expression in single transfection showed significant upregulation, as expected. Co-transfected group also maintained mortalin levels unlike the decrease seen in HIV-1 Tat-transfected cells, as analzsed by RT-PCR (Fig. 3a) and Western blotting (Fig. 3b, c), thus corroborating data in Fig. 2. However, probing for HIV-1 Tat leads to a surprising observation, that while there was a clear and concise band in HIV-1 Tat-transfected cells, the same was absent in the co-transfected group (Fig. 3c). To determine the above observation whether driven by exogenously overexpressed mortalin, we immunostained the transfected PDA with anti-V5 and anti-HIV-1 Tat antibodies to visualize the expression of both these proteins (Fig. 3d). As in Fig. 3c, the absence of Tat protein in cotransfected lane is not the result of cell death due to overexpression of mortalin with Tat which is clearly verified from these immmunographs, as Fig. 3d clearly shows the expression of exogenous mortalin. This suggested that cotransfection of mortalin affected the HIV-1 Tat protein but did not alter the cell number, thus explaining why there was no cell toxicity and death in cotransfection. However, we also observed few cells positive with HIV-1 Tat in cotransfected cells (FigS.6). In order to investigate the reasons behind Tat disappearance in co-transfected cells, we checked for proteosomal-mediated Tat degradation. After 12 h of transfection, cells were treated with proteosomal inhibitor MG132. This led to the restoration of Tat band in co-transfected group (Fig. 3e). This data confirmed that overexpression of mortalin resulted in degradation of Tat. To gain deeper understanding of the mechanism behind Tat degradation, we used a bioinformatics simulation tool to predict interaction between mortalin and HIV-1 Tat. As shown in Fig. 3f, Protein Data Bank (PDB) structures of mortalin (3N8E) and Tat (1TIV) were obtained and simulated in Clus-pro online protein docking tool, followed by visualization of the dock structure using pymol visualizing tool. This predicted the direct interaction of HIV-1 Tat and mortalin. Next, to validate these in silico findings with in vitro assays, we performed coimmunoprecipitation assay, and whole cell lysate from PDA after 24 h transfection was isolated. Lysates from HIV-1 Tat and co-transfected cells were precipitated with anti-mortalin antibody and probed for anti-HIV-1 Tat and anti-mortalin antibodies (Fig. 3g). While HIV-1 Tat showed a clear band (Fig. 3g, third lane), the mortalin band was faint, indicating that mortalin interacts with HIV-1 Tat, but its expression is reduced in presence of Tat. In the cotransfected group (Fig. 3g, last lane), mortalin is strongly precipitated with Tat, however, Tat is observed as a very faint band indicating its degradation by overexpressed mortalin. In reverse coimmunoprecipitation, cells were isolated from the transfected PDA and precipitated with anti-HIV-1 Tat antibody, and blots were probed with anti-mortalin and anti-HIV-1 Tat, which showed the similar interaction (Fig. 3h).

### Mortalin rescued the mitochondrial health following HIV-1 Tat-mediated toxicity

**Fig. 4:**
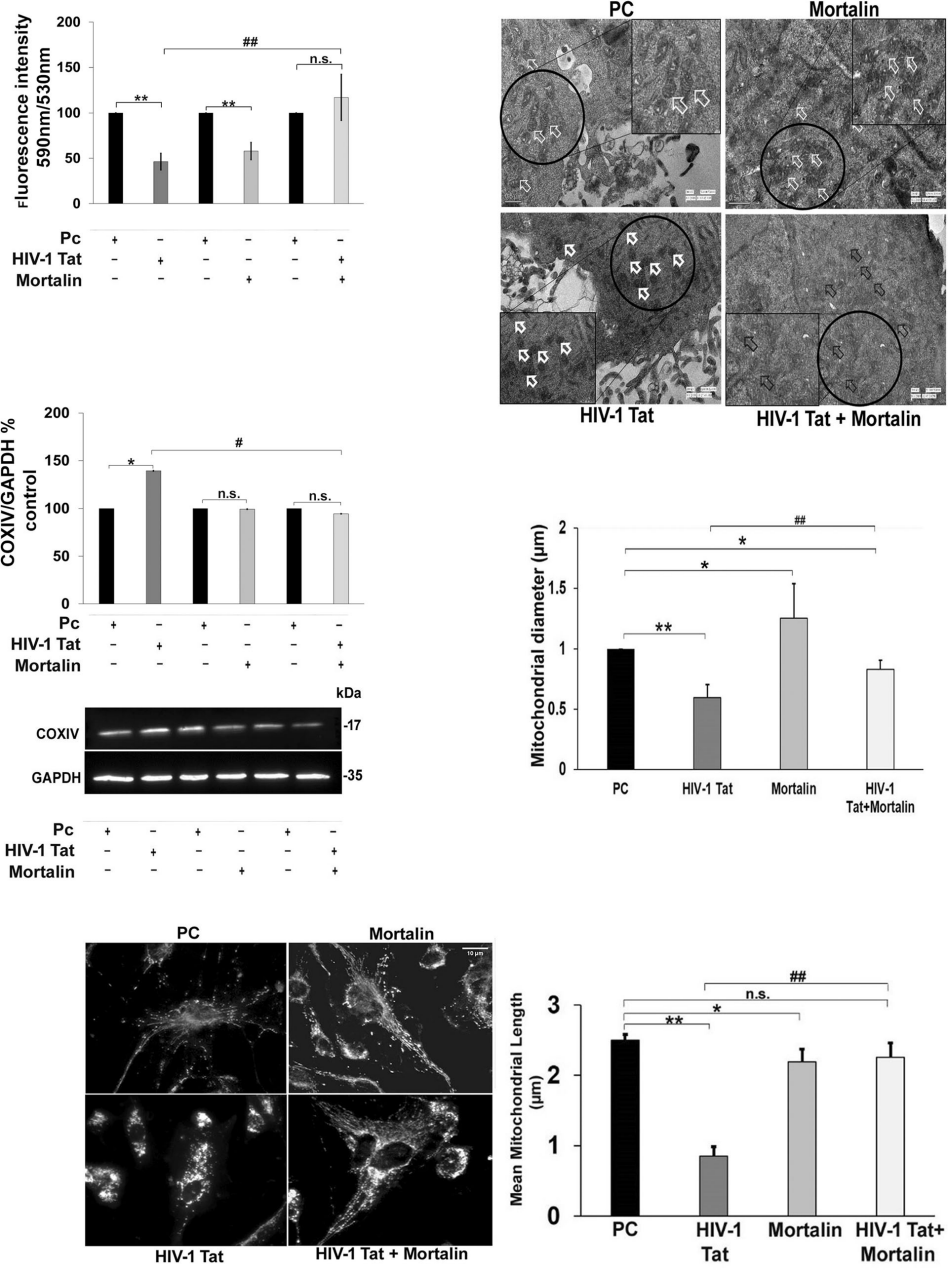
Overexpression of mortalin reduces the HIV-1 Tat mediated mitochondrial dysfunction and fragmentation. **a**) Percentage change in mitochondrial membrane potential (mean fluorscence intensity of JC-1 aggregates/JC-1 monomers) in indicated transfected groups after 24 h, as analyzed by fluorescence estimation (*n* = 5). **b**, **c**) Percentage change and representative western blot showing change in COXIV expression in all transfected PDA groups. GAPDH was used as the loading control (*n* = 5). **d**) Representative images of mitchondria labelled with mitotrackerRED after 24 h of transfection with indicated plasmids (*n* = 4). **e**) Quantitative analysis of mitochondrial length stained with mitotrackerRED (*n*=4). **f**) Representative electron microscopic images showing mitochondrial morphology in transfected groups. Arrows indicate regions showing differently shaped mitochondria (*n*=3). **g**) Quantitative analysis of mitochondrial diameter of electron microscopic images in transfected cell (*n* = 3). All data represent mean ± standard deviation, from independent experiments (*n* stands for number of independent experiments, *p< 0.05, **p< 0.005 with respect to control, #*p*< 0.05, ##*p*< 0.005 with respect to HIV-1 Tat and cotransfected group (PC stands for plasmid control and PC+PC is used as plasmid control in cotransfected cells)

We next sought to examine the role of mortalin in HIV-1 Tat-mediated toxicity. Several studies suggest the direct and indirect influence of Tat in altering mitochondrial morphology and health, thereby inducing detrimental changes in its biochemical functions [15, 16]. In order to decipher the effect of mortalin in transfected PDA, we first measured the mitochondrial membrane potential in mortalin, HIV-1 Tat, and co-transfected groups, and found that cotransfected cells showed intact membrane potential with no significant difference compared with empty vector transfected cells, whereas HIV-1 Tat transfection showed almost 50% reduction in membrane potential (Fig. 4a). This indicated that HIV-1 Tat-induced destabilization in mitochondrial membrane potential was restored by mortalin. The cytochrome c oxidase or COXIV is regarded as a major regulator of oxidative phosphorylation and exerts tight regulation on mitochondrial functions and its dysregulation leads to compromised mitochondrial membrane potential, hence we sought to investigate the COXIV at protein level in the transfected cells, and we observed that Tat transfection increased the COXIV levels compared with control. Cotransfected cells did not show any noticeable changes, again suggesting that overexpression of mortalin in presence of Tat equilibrates COXIV expression (Fig. 4b, c). In order to assess the effect of cotransfection on Tat mediated alteration in mitochondrial morphology through dysregulation of the fusion and fission machinery, we performed mitochondrial imaging in transfected cells labelled with mitotrackerRED. The microscopic images of HIV-1 Tat transfected cells showed increased mitochondrial fragmentation, as several short, fragmented mitochondria were seen (Fig. 4d). Unlike Tat transfected cell, mitochondrial morphology in cotransfected cells were more branched, elongated, and comparable with vector control images. Marked reduction in mitochondrial length was observed in Tat transfected cells as compared with empty vector, however the cotransfected cells showed significant change as compared with Tat (Fig. 4e). To gain in-depth details of ultrastructure alterations in mitochondria, we relied on electron microscopy. The analyses of electron microscopy of transfected PDA revealed dense amorphous structure of cristae with smaller, circular mitochondria in Tat transfected cells only. However, cotransfected cells depicted distinct tubular cristae as compared with Tat alone (Fig. 4f). Indeed, Tat promotes significant reduction in mitochondrial diameter approximately from 1 to 0.4 μm, while cotransfection or overexpressed mortalin showed increased mitochondrial diameter as compared with Tat (Fig. 4g).In addition, overexpressed mortalin alone also showed the destabilized mitochondrial membrane potential, however these damaging effects were not observed in mitochondrial morphology visualized by mitotrackerRED staining and electron microscopy (Fig. 4a, e, g).

### Mortalin regulates extracellular ATP release in HIV-1 Tat transfected PDA

**Fig. 5:**
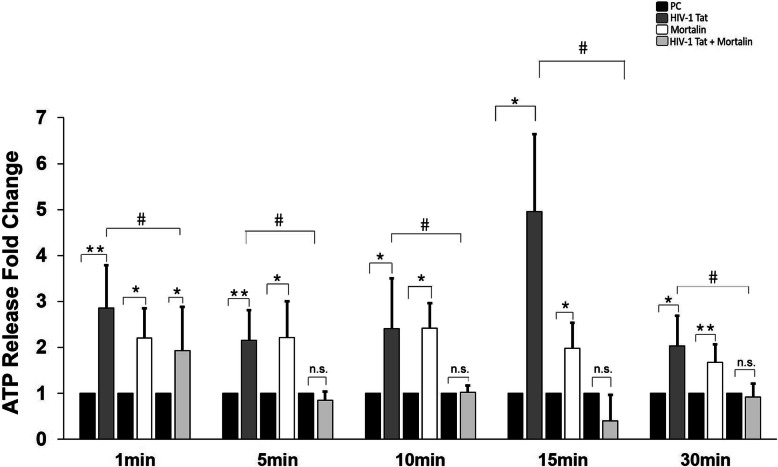
Regulation of extracellular ATP burst in cotransfected astrocytes. Fold change showing extracellular ATP release in PDA after 24 h of transfection with HIV-1 Tat, mortalin, and cotransfection (mortalin and HIV-1 Tat), and empty vectors were taken as control (*n*=5). All data represents mean ± standard deviation, from independent experiments (*n* stands for number of independent experiment), *p< 0.05, **p< 0.005 with respect to control, #*p*< 0.05 with respect to HIV-1 Tat and cotransfected group. Color shades represent black blocks which are for plasmid control (PC), dark grey which are HIV-1 Tat, white blocks which are overexpressed mortalin, and light grey which are cotransfected with mortalin and HIV-1 Tat

In order to investigate the direct damage due to HIV-1 Tat on mitochondrial function, we measured the extracellular ATP release in variously transfected PDA, at different time intervals, as a test for mitochondrial function. Post 24 h of transfection in PDA, we found that extracellular ATP was significantly lower in the cotransfected cells at all-time points, whereas the ATP release was highly uncontrolled in Tat transfected cells (Fig. 5). ATP release at 15 min was consistently found to be the highest in the Tat group, compared to the other groups. Together, this and above data indicate that cotransfection of mortalin with Tat maintain mitochondrial health and can also regulate the ATP release from PDA.

### Decrease in release of inflammatory cytokines by mortalin

**Fig. 6:**
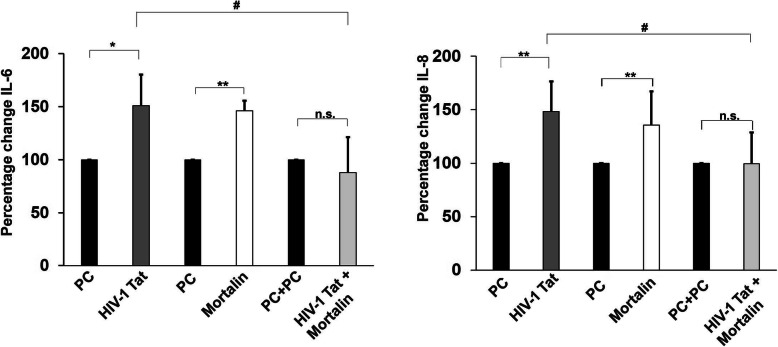
Levels of released cytokines in transfected PDA. Percentage change showing levels of released IL-6 and IL-8 in supernatant of PDA post 24 h of transfection with indicated plasmids (*n*=5). All data represents mean ± standard deviation, from independent experiments ( *n* stands for number of independent experiment, *p< 0.05, **p< 0.005 with respect to control, #*p*< 0.05 with respect to HIV-1 Tat and cotransfected group (PC stands for plasmid control, and PC+PC is used as plasmid control in cotransfected cells)

The abnormally hyper activation of immune system has been widely reported in viral infection including HIV-1 and significantly correlated with the severity of the disease [40, 41]. Previously, it has been shown that HIV-1 Tat induces the expression of pro-inflammatory molecules like IL-6 and IL-8 in both human monocytes and dendritic cells of HIV-1 patients [13]. In this study, we evaluated the extracellular levels of IL-6 and IL-8 in the supernatants of variously transfected PDA (Fig. 6). We found that Tat transfected cells demonstrated significant increase in release of IL-6 and IL-8 compared with control cells. We also detected the levels of IL-1β, IL-12p70, and TNF-α in the supernatants of same transfected cells, however, no significant change was observed (data not shown). Cotransfection led to significantly decreased release of IL-6 and IL-8, which was comparable to control cells. This data suggested that Tat induced release of pro-inflammatory cytokines is regulated by mortalin.

### Mortalin mediated rescue of ROS, and NF-kB-p65 signalling activated by HIV-1 Tat

**Fig. 7:**
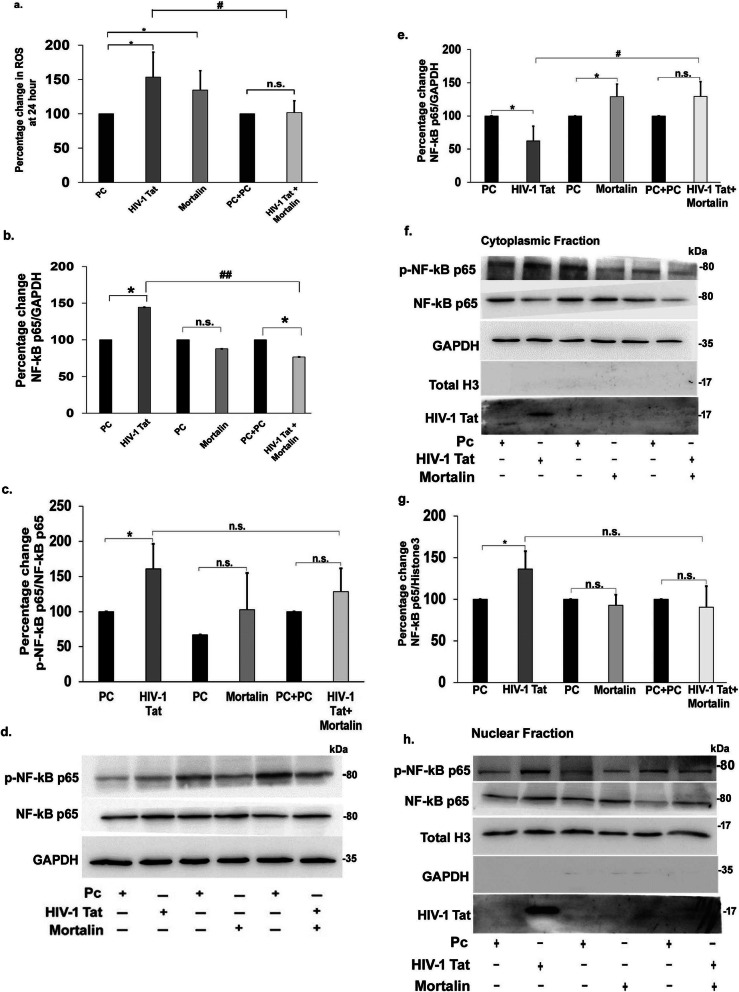
Assessment of ROS production and activation of NF-kB-p65. **a**) Percentage change showing levels of ROS in PDA post 24 h transfection with indicated plasmids (*n*=4). **b**) Percentage change showing the levels of total NF-kB-p65 in post transfected PDA (*n* = 5). **c**) Representative change in the expression of phosphorylated-NF-kB-p65 in all transfected PDA groups is represented in the graph (*n*=5). **d**) Representative western blot showing change in expression of NF-kB-p65 and phosphorylated NF-kB-p65 in transfected PDA (*n*=5). GAPDH was used as the loading control. **e**) Percentage change of the cytoplasmic fraction in PDA post 24 h transfection showing the levels of Nf-kB-p65 (*n* = 4). **f**) Expression levels of cytoplasmic Nf-kB-p65 were evaluated by represented western blots. GAPDH was used as the internal control (*n*=4). **g**) Percentage change of the nuclear fraction in PDA post 24h transfection showing the levels of Nf-kB-p65 (*n*=4). **h**) Expression levels of nuclear Nf-kB-p65 were evaluated by represented western blots.Total histone3 was used as the internal control (*n* =4). All data represent mean ± standard deviation from independent experiments (*n* stands for number of independent experiment), *p< 0.05, with respect to control, #*p*< 0.05, ##*p*< 0.005 with respect to HIV-1 Tat and cotransfected group (PC stands for plasmid control, and PC+PC is used as plasmid control in cotransfected cells)

The dysregulation in the balance between the rate of production and the clearance of reactive oxygen species (ROS) leads to oxidative stress in cells, thus affecting cellular homeostasis, leading to the development of pathophysiological conditions in neurodegenerative diseases [42]. Here, we also tried to detect the levels of cellular ROS in the transfected PDA, and we found significant increase in cellular ROS in Tat or mortalin single-transfected groups, but the ROS levels were reduced by almost half in the cotransfected cells, compared with Tat transfection, as measured by DCFDA fluorescence stain (Fig. 7a). Accumulation of ROS beyond its basal level activates downstream signaling of NF-kB. We next analyzed the protein levels of NF-kB-p65 and phosphorylated NF-kB-p65 in the transfected cells (Fig. 7d). The percentage change in the expression of NF-kB-p65 showed almost 50% upregulation in Tat transfected cells, compared with control. However, the expression of NF-kB-p65 was significantly downregulated in cotransfected cells as compared with Tat (Fig. 7b). These elevated levels of NF-kB-p-65 were significantly correlated with the phosphorylation of NF-kB-p65 in the Tat transfected cells, however no change was observed in the levels of phosphorylated NF-kB-p65 in cotransfected cells (Fig. 7c). Translocation of NF-kB plays an important role in the inflammation, hence, we next examined the nucleocytoplasmic translocation of NF-kB-p65 by western blot analysis in cytoplasmic and nuclear extracts in transfected PDA (Fig. 7f, h). We observed significant decrease in cytoplasmic NF-kB-p65 expression in Tat transfected cells, and a marked increase in nuclear translocation was observed, but no translocation was noted in cotransfected cells, neither in cytoplasmic (Fig. 7e), nor in the nuclear extract (Fig. 7g). Together, from this and the above data, we strongly believe that Tat is degraded before it can activate inflammatory signalling.

### Mortalin reduces extracellular glutamate release and indirectly rescues HIV-1 Tat-mediated neuronal death

**Fig. 8:**
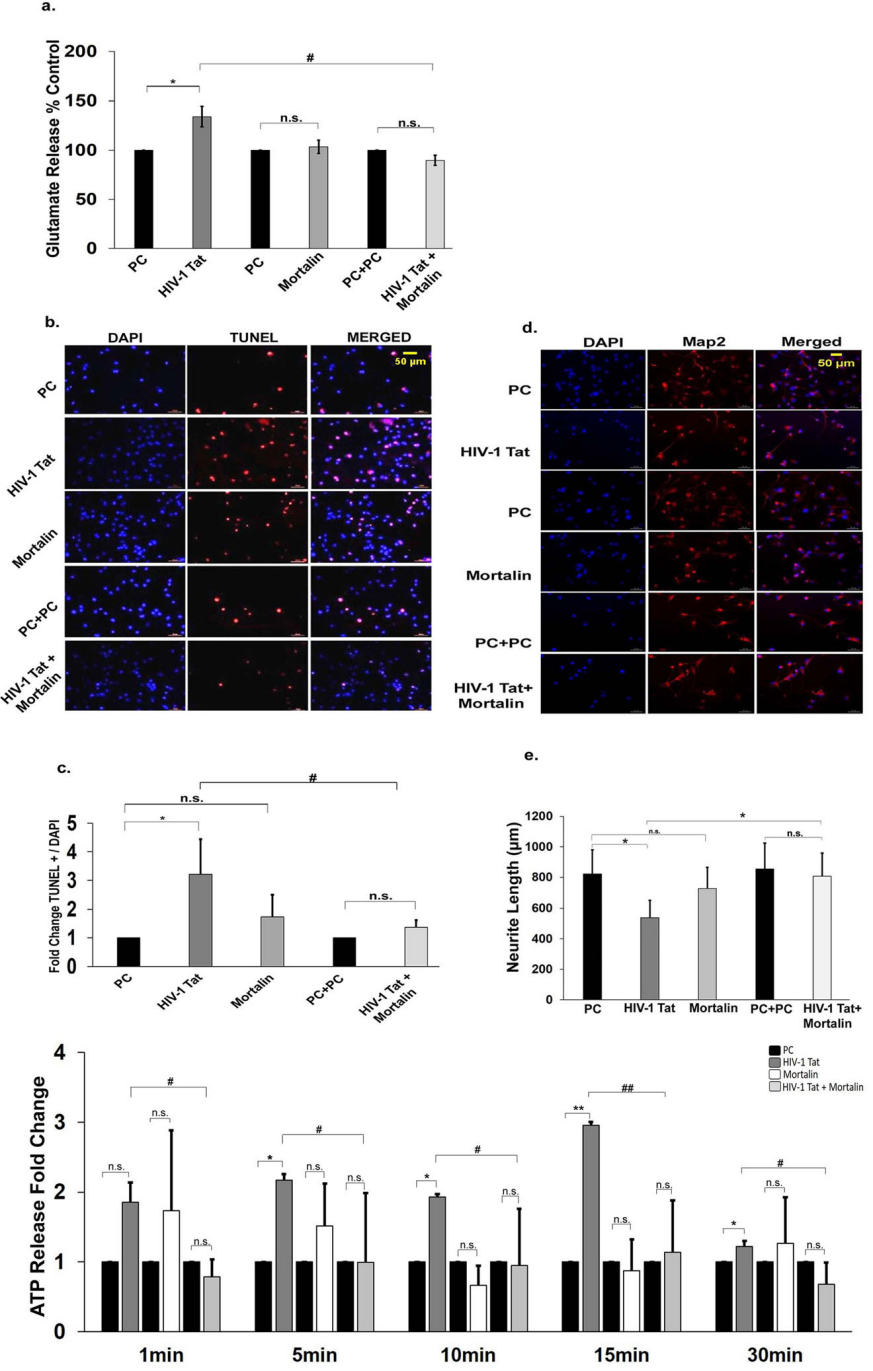
Assessment of astrocytic mediated neuronal damage. **a**) Percentage change in the extracellular release of glutamate in transfected PDA (*n* = 4). **b**) Immunofluorescence images of neurons post 24 h of treatment with astrocytic condition media (ACM) transfected for 24 h, stained with TUNEL (red dots), and counterstained with DAPI for nuclei identification, Scale bar indicates 50 μm. **c**) The TUNEL positive cells were counted from five random fields from each set and divided by the total number of cell in the respective field, and fold change represents the TUNEL positive neurons treated with ACM (*n*=3). **d**) Immunofluorescence images of neurons post 24 h of treatment with astrocytic condition media of transfected PDA, neurons were labelled with anti-MAP2 antibody in red for the detection of neurite outgrowth and counterstain with DAPI for nuclei identificatiom, Scale bar is 50 μm. **e**) Change in the neurites length in treated neurons with ACM was determined using imageJ, and five images were randomly selected from each treatment set (*n* = 3). **f**) Fold change showing extracellular ATP release in treated neurons with ACM after 24 h of transfection with various plasmids (indicated in different colors, shown in key). Color shades represent black blocks which are for plasmid control (PC), dark grey which are HIV-1 Tat, white blocks which are overexpressed mortalin, and light grey which are cotransfectd with mortalin and HIV-1 Tat (n = 3). All data represents mean ± standard devitaion, from independent experiments (n stands for number of independent experiment), *p< 0.05, **p< 0.005 with respect to control, #*p*< 0.05, ##*p*< 0.005 with respect to HIV-1 Tat and cotransfected group (PC stands for plasmid control, and PC+PC is used as plasmid control in cotransfected cells)

Astrocytes are known to tightly regulate the glutamate levels in extracellular milieu, thus protecting neurons from glutamate excitotoxicity mediated death [43]. Under HIV infected conditions, this regulation is disrupted leading to excessive accumulation of glutamate in synaptic clefts. Here, we checked the effect of mortalin on astrocyte mediated neuronal death induced by Tat. Extracellular glutamate levels in Tat transfected PDA were found to be significantly increased (approximately 30%) (Fig. 8a), whereas it showed marked reduction in the cotransfected group. To assess indirect neuronal death, neurons supplemented by conditioned media from transfected astrocytes were analyzed for in situ cell death using a highly sensitive apoptosis detection assay, the TUNEL assay. TUNEL assay revealed approximately 3-fold increase in neuronal death upon treatment with Tat transfected astrocyte conditioned media (ACM) as compared to control (Fig. 8b, c). The neurons cotreated with Tat and mortalin supernatant were successfully rescued from astrocytic mediated neuronal death induced by Tat. To determine the effect of Tat and mortalin on neurite outgrowth, the neurons were treated with ACM for 24 h. Neurons were stained with Map2, and neurite length was measured. Significant reduction in neurite length was observed in Tat treated neurons and cotreated neurons showed increase neurite outgrowth (Fig. 8d, e). The increased extracellular ATP activates further downstream signalling and also increase the neuronal death, and we next detected the indirect release of extracellular ATP in ACM treated neurons and found it to be significantly reduced in cotransfected group, compared to HIV-1 Tat or empty vector (Fig. 8f).

### Endogenous mortalin levels are reduced in brains of HIV-1-infected patients

**Fig. 9:**
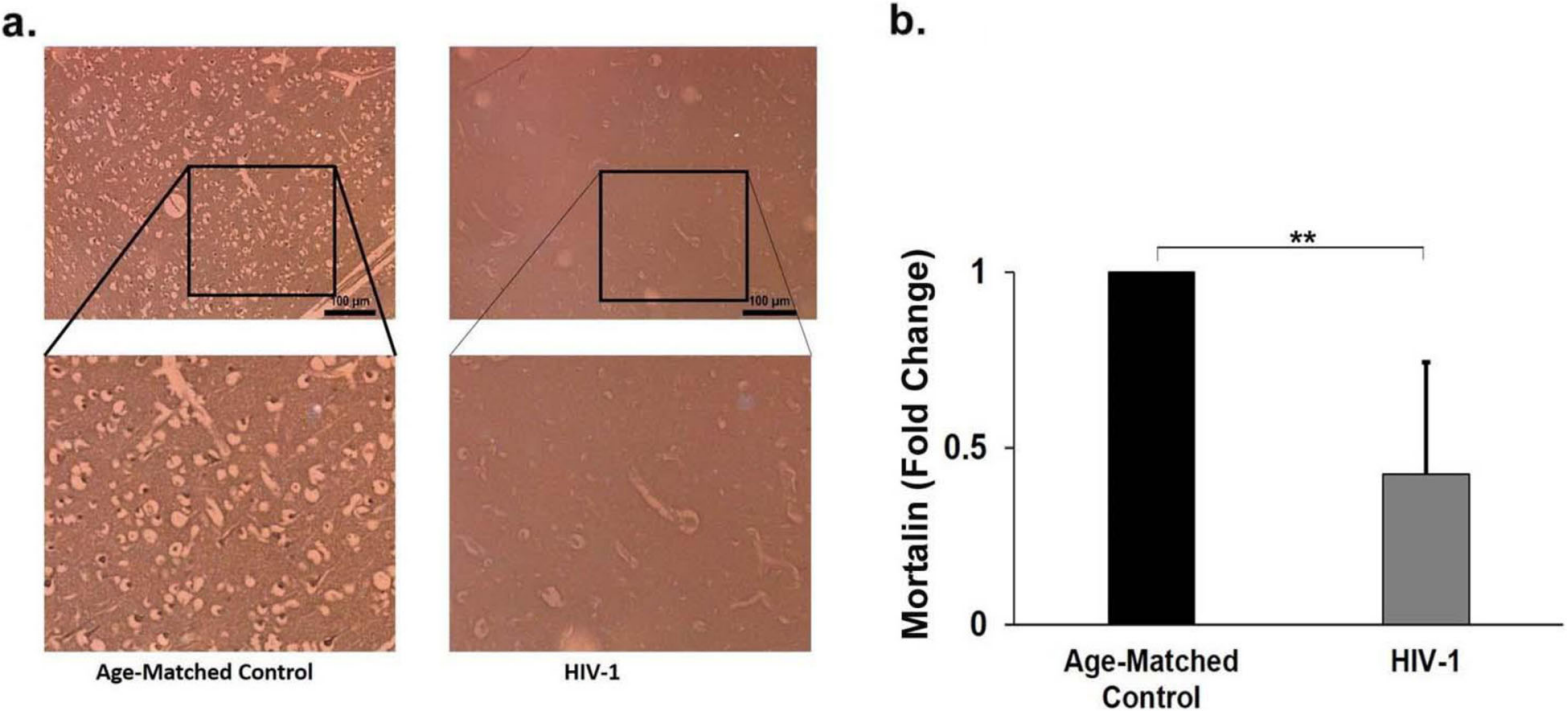
Endogenous mortalin is severely reduced in autopsy samples of HIV-1 positive adult brain sections. a) Representative immunohistochemical image of frontal cortex region of adult human brain sections showing expression of mortain (dark brown cells). Section stained in right panel is from a HIV-1 positive individual and left panel is an age matched control, lower panels are the magnified views of indicated regions (n=3). b) Bar graph representd the fold change of mortalin in frontal cortex region of controls and HIV-1 infected individuals. The data represents from three age-matched controls and HIV-1-infected brain sections. Data represented as mean ± Standard Deviation. **P<0.005 as compared with control

To validate our in vitro findings, we examined the levels of mortalin in human autopsy samples of HIV-1 patients who did not have any other opportunistic disease at the time of death. Immunohistochemistry (IHC) revealed severe downregulation of mortalin in the HIV-1 positive sections, compared to their age-matched non-HIV-1 controls in the frontal region of the brain (Fig. 9a, b). IHC was also performed in the hippocampal sections of HIV-1 positive and age-matched control individuals which corroborated the above findings (data not shown).

## Discussion

Although cART has successfully reduced the viral load to undetectable level and also increase the life expectancy of the HIV infected patients, but it has not completely cured the AIDS patients, as HIV-1 continues to thrive in certain niche areas where cART cannot access the viruses. While HIV can cross the Blood-Brain Barrier, cART drugs are unable to do so, making the brain a “safe haven” for HIV-1. Not surprisingly, more than 50% of HIV-1-infected patients develop HIV-1 associated neurological disorder despite being treated with cART.

Amongst other HIV-1 proteins, HIV-1 Tat causes neuroinflammation and neurodegeneration. Tat initiates a destructive cycle in the brain involving collapse of mitochondrial permeability and high calcium influx, leading to excitotoxicity [15, 43, 44]. These pathological events in the brain are facilitated through infecting the astroglia and microglia cells which continuously harbor the virus for long-term and are critically affected by Tat. Unlike other HIV viral proteins, Tat is detected in the CSF of HIV-1 patients on cART, and reveal its detrimental role in brain cells. In Tat mediated neuronal damage, astrocytes emerged as a mediator in inducing direct and indirect death of brain cells. During HIV infection, the infected cells, including astrocytes, release viral proteins such as Tat and Nef, and other neurotoxins which perturbs the neuro-glia interaction and leads to dysregulated neural circuits, synaptic damage, and dendritic loss.

While several host factors aggravate or diminish HIV-1 mediated effects in the body, we focused our study on mortalin, a member of the heat shock protein 70 (HSP70) family. Recent studies have shown that HSP70 expression was inversely correlated with viral replication [45], indicating that this could play an important role in the progression and prevention of the disease manifestation. HIV-1 was also found to decrease the levels of HSP70, and this is also corroborated by our study. Interestingly, our data suggest that mortalin interaction with HIV-1 Tat leads to degradation of the latter, thus critically neutralizing the effects of HIV-1 Tat on the host.

Earlier research has shown the downregulation of mortalin in the autopsy samples of Parkinson’s patients [30]. The transgenic AD-mouse model system also correlated the reduction of mortalin with AD diseased patients [32]. In contrast, overexpression of mortalin in the AD model system successfully reduced the mitochondrial fragmentation and cell death. However, the efforts to correlate the progression of disease with the expression of mortalin remained inconclusive [46]. We hence decided to undertake this study to understand if mortalin has any role in HIV-1 neuropathogenesis.

Our data demonstrated mortalin levels to be downregulated both in autopsy sections of HIV-1 seropositive patients and in Tat transfected astrocytes. This suggests the viral defence mechanism affects the expression of mortalin, which might in turn intrude the HIV replication. However, cotransfection of mortalin with HIV-1 Tat showed that Tat is degraded in the presence of excess mortalin, strongly suggesting that the stoichiometric ratio of both these proteins in the cells determine whether the effects of HIV-1 Tat or mortalin will be predominant over the other. Moreover, cotransfection of mutated Tat with mortalin does not downregulates the expression of Tat (FigS.4). This suggest mortalin-specific binding with wild-type Tat-B only.

Basal expression of mortalin in cell helps in maintaining mitochondrial integrity, import and export of the various protein from in and out to the mitochondria, and also participates in protein folding. Induction of mortalin expression in mild stress condition offers protection against oxidative stress and ischemic injury to cell as an adaptive response, whereas the highly elevated levels of mortalin in cancer cell promote cell survival by inhibiting the p53 function [34, 47]. These contrasting conditions of cell explain the stoichiometry of mortalin. Here, in our study as well, the upregulation of mortalin reduces the Tat expression, thus indirectly preventing Tat mediated cell death.

In most of our results (Fig. 4a, Fig. 6a, and Fig. 7a), we found that overexpression of mortalin alone led to decline in mitochondrial membrane potential and increase in proinflammatory cytokines at 24 h. However, when we examined the ultrastructure of mitochondria and mitochondrial fragmentation assay, we did not notice any damage to the mitochondria.

A cell-based functional knockdown screen study found mortalin to be a critical regulator of mitochondrial dynamics. Knockdown of mortalin led to mitochondrial loss in neuronal synapses. Similar effects were also reported in HIV-1 Tat infected cells, in which profound mitochondrial fragmentation, dysregulated fusion, and fission machinery were noticed in neurons [16, 48]. Knockdown of mortalin in PDA also shows significant downregulation in mitochondrial membrane potential (FigS.3.b) and also affects the mitochondrial length and mitochondrial morphology significantly (FigS.3.c, d).

Mitochondria have also been reported to play a major role in neuronal survival by various mechanisms, such as calcium homeostasis, control in ROS production, and energy production in the form of ATP. Thus, we focused on the effect of mortalin in controlling the damage to mitochondrial health by HIV-1 Tat. Our data showed that HIV-1 Tat mediated effect on membrane potential was rescued by overexpressed mortalin, including reduced levels of cytochrome c, a major regulator of oxidative phosphorylation. Overexpression of mortalin was also found to restore the balance between fusion and fission machinery, leading to lesser mitochondrial fragmentation. Alteration in the ultrastructure of mitochondrial cristae are usually associated with several diseases including HIV, while good mitochondrial health is correlated with clear and intact cristae. Our electron microscopy data confirmed the successful rescue of mitochondrial health—identified from its membrane potential, regulated cytochrome c oxidase IV levels of proteins, and intact mitochondrial morphology. Together, these results suggest that the rescue in mitochondrial morphodynamics and its function may be due to the degradation of Tat following mortalin binding before Tat can induce its deleterious effect.

Beside mitochondrial health, mortalin elicits multifunctional mechanism of protection against HIV-1 Tat. In addition, few events generally get activated as a defence mechanism of cell in response to foreign pathogen. In the HIV-1 condition, astrocytic perturbation leads to astrogliosis, a hallmark of pathophysiological condition. The alleviated levels of glial fibrillary acidic protein (GFAP) are extensively correlated in reactive astrocytes. Here, in our study also, we observed significant increase in the expression of GFAP in Tat transfected PDA which was successfully rescued in cotransfected PDA (FigS.5 a, b). The microscopic images (FigS.5.c) confirmed the GFAP aggregates formed in Tat transfected cells only, which was corroborated by fluorescence intensity (FigS.5.d). The negligible change at GFAP expression in cotransfected cells was found. HIV-1 Tat mediated astrogliosis also releases inflammatory cytokines and other inflammatory factors. The early and enhanced ROS generation was noted which was rescued by mortalin, and this confers that cotransfection of mortalin with Tat is not only degrading the Tat protein but simultaneously deactivating/reducing the generation of ROS.

In our study, we observed that transfection with HIV-1 Tat increased the glutamate and ATP release in supernatant and also resulted in neuronal damage by an indirect mechanism. We observed that cotransfection in astrocytes resulted in decreased neuronal death and controlling the ATP release from neurons. Indirect neuronal death mediated by astrocytes is the hallmark of neuropathogenesis in HIV-1. Loss in astrocytic support to neuron promotes accumulation of glutamate in the synapses leading to excitotoxicity and decreased dendritic and synaptic density, which constitute the pathological features and associated cognitive dysfunction in the infected patient.

## Conclusion

To summarize, we have identified a novel role of mortalin in HIV-1 Tat transfected astrocytes. In our study, mortalin binds to Tat in astrocytes and degrades Tat, making Tat unavailable for cellular toxicity. This rescued the cell from Tat mediated detrimental effects on astrocytes and also reduces the indirect neuronal death. This study suggests a therapeutic role of mortalin against HIV-1 Tat induced neuronal damage and advocates for further studies in brain organoid and other in vivo models.

### Statistical analysis

All experiments were performed with biological as well as technical replicates and performed four to five times independently. “n” indicates the number of independent experiments. Statistical significance between control and transfected groups was computed using Student’s *t* test. *P* < 0.05 was taken as statistically significant.

## Supplementary information

**Additional file 1 **: **Fig.S1**. Expression of mortalin in human fetal brain-derived cells. Representative Immunocytochemistry images of human progenitor cells (upper panel) stained with anti-mortalin (red) and anti-nestin (green0 and merged depicting co-localization of these proteins. Neurons differentiated from progenitor cells were stained with anti-mortalin (red) and anti-Map2 (green) and merged depicting the co-localization of these proteins. Scale bar 50 μm (*n* = 3). **Fig.S2**. Transfection efficieny of plasmid in PDA. PDA transfected with PEGFP plasmid for 24h, depicting 20-25% transfection efficiency. **Fig.S3**. Knockdown of mortalin in PDA damages the mitochondrial health. a) Western blot represents the knockdown of mortalin in PDA. b) Fold change in mitochondrial membrane potential after 24h of transfection, (*n*=3). c) Quantitative analysis of mitochondrial length stained with mitotrackerRED (*n* = 3). d) MitotrackerRED stained images of PDA after 24h of trasnfection, Scale bar 10μm (*n* = 3). Data represent mean±standard deviation, from independent experiments (n stands for number of independent experiment, **p< 0.005 with respect to control. **Fig.S4**. Expression of mutant Tat. Western blots showing the expression of Tat in cotransfected PDA. Abbreviations are: CC (cysteine-cysteine) and SC (serine-cysteine) M-Mortalin and T-Tat. **Fig.S5**. GFAP expression and aggregation in PDA. a) Fold change GFAP in transfected astrocytes (*n* = 4). b) Representative western blot of GFAP at protein level (*n* =4). c) Immunocytochemistry of GFAP (green) counterstained with DAPI, showing aggregates of intermediate filaments. Scale bar 20μm. d) Fold change in the mean fluorescence intensity of GFAP (*n* = 3). All data represent mean±standard deviation, from independent experiments*p< 0.05, **p< 0.005 with respect to control, #*p*< 0.05, ##*p*<0.005 with respect to HIV-1 Tat and cotransfected group. **Fig. S6**. Expression of HIV-1 Tat in cotransfected PDA. Representative immunofluorescence images of cotransfected PDA post 24h of transfection, showed the expression of Tat and exogenous mortalin tagged with V5, Scale bar 20 μm (*n* = 3).

## Data Availability

All datasets generated and/or analyzed in this study are available from the corresponding author on reasonable request.

## Abbreviations

CNS: Central nervous system
ROS: Reactive oxygen species
BBB: Blood-brain barrier
HIV-1: Human immunodeficiency virus
Tat: Transactivator of Transcription
ATP: Adenosine triphosphate
Drp1: Dynamin-related protein-1
Mfn2: Mitofusin-2
PCR: Polymerase chain reaction
PDA: Progenitor-derived astrocytes
DCFDA: 2′, 7′-dichlorofluorescin diacetate
IL-6: Interleukin-6
IL-8: Interleukin-8
IL-12p70: Interleukin-12p70
TNF-α: Tumour necrosis factor-alpha
ACM: Astrocytic conditioned media
