# Supplementary material for: Novel role of mortalin in attenuating HIV-1 Tat-mediated astrogliosis
Source: J Neuroinflammation. 2020 Sep 20;17:276. doi: 10.1186/s12974-020-01912-3 (PMC7504834; doi:10.1186/s12974-020-01912-3)
Supplement: Supplementary file 1 — Additional file 1 : Fig.S1. Expression of mortalin in human fetal brain-derived cells. Representative Immunocytochemistry images of human progenitor cells (upper panel) stained with anti-mortalin (red) and anti-nestin (green0 and merged depicting co-localization of these proteins. Neurons differentiated from progenitor cells were stained with anti-mortalin (red) and anti-Map2 (green) and merged depicting the co-localization of these proteins. Scale bar 50 μm (n = 3). Fig.S2. Transfection efficieny of plasmid in PDA. PDA transfected with PEGFP plasmid for 24h, depicting 20-25% transfection efficiency. Fig.S3. Knockdown of mortalin in PDA damages the mitochondrial health. a) Western blot represents the knockdown of mortalin in PDA. b) Fold change in mitochondrial membrane potential after 24h of transfection, (n=3). c) Quantitative analysis of mitochondrial length stained with mitotrackerRED (n = 3). d) MitotrackerRED stained images of PDA after 24h of trasnfection, Scale bar 10μm (n = 3). Data represent mean±standard deviation, from independent experiments (n stands for number of independent experiment, **p< 0.005 with respect to control. Fig.S4. Expression of mutant Tat. Western blots showing the expression of Tat in cotransfected PDA. Abbreviations are: CC (cysteine-cysteine) and SC (serine-cysteine) M-Mortalin and T-Tat. Fig.S5. GFAP expression and aggregation in PDA. a) Fold change GFAP in transfected astrocytes (n = 4). b) Representative western blot of GFAP at protein level (n =4). c) Immunocytochemistry of GFAP (green) counterstained with DAPI, showing aggregates of intermediate filaments. Scale bar 20μm. d) Fold change in the mean fluorescence intensity of GFAP (n = 3). All data represent mean±standard deviation, from independent experiments*p< 0.05, **p< 0.005 with respect to control, #p< 0.05, ##p<0.005 with respect to HIV-1 Tat and cotransfected group. Fig. S6. Expression of HIV-1 Tat in cotransfected PDA. Representative immunofluorescence images of cotrans [file 12974_2020_1912_MOESM1_ESM.pdf]

FigS.1

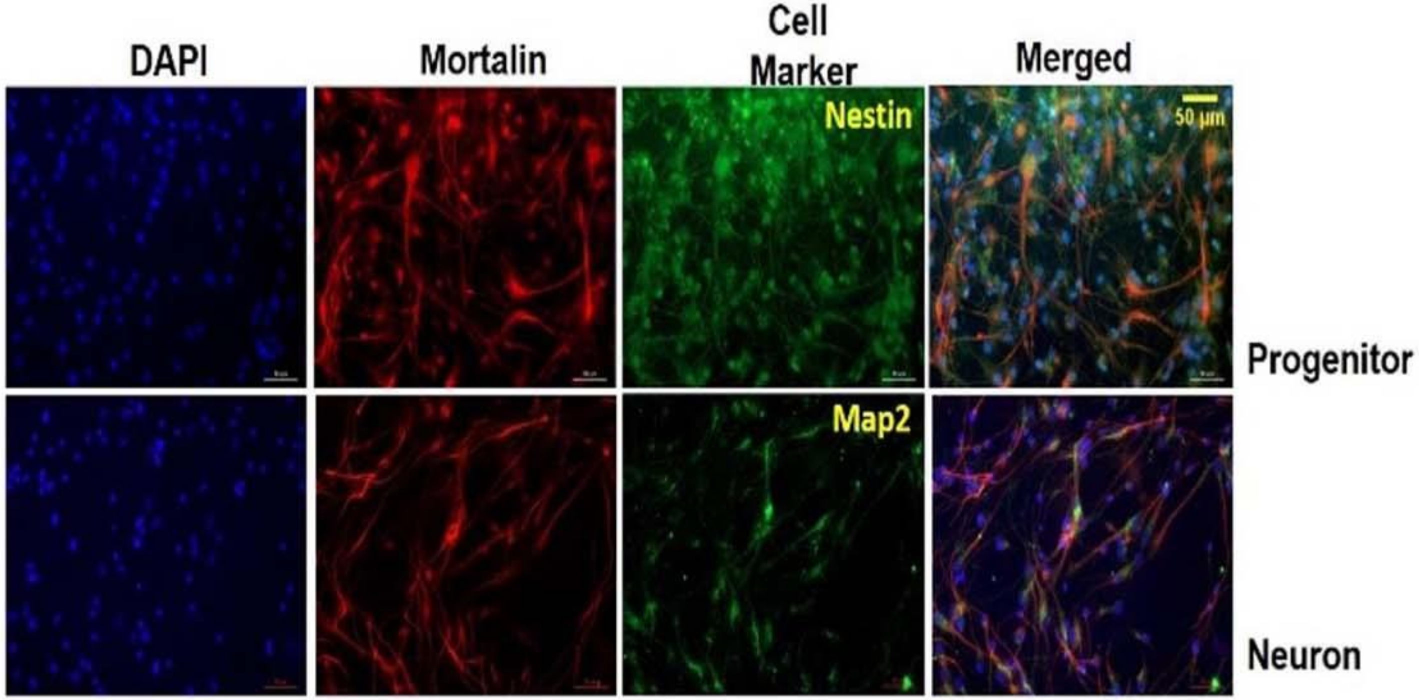

FigS.1) Expression of mortalin in human fetal brain derived cells. Representative immunocytochemistry images of human progenitor cells (upper panel) were stained with anti-mortalin in red and anti-nestin in green and merged depicting co-localization of mortalin and nestin. Scale bar, 50μm (n=3). Human neuronal cells differentiated from human fetal brain derived progenitor were stained with anti-mortalin in red and anti-Map2 in green and merged depicting the co-localization of both these protein. Scale bar 50μm (n=3)

FigS.2

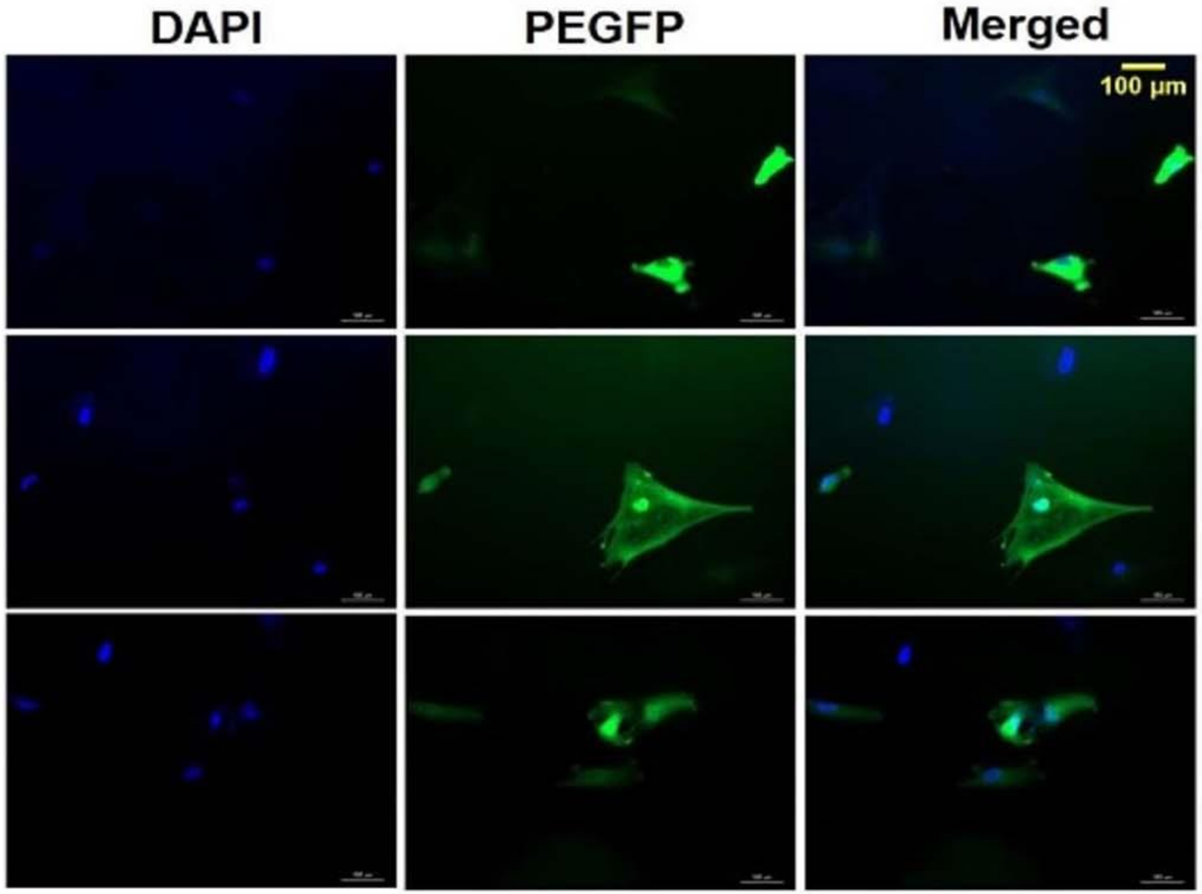

FigS.2) Transfection efficiency of plasmid in progenitor derived astrocytes. PDA transfected with PEGFP plasmid for 24h, depicting 20-25% of transfection efficiency in progenitor derived astrocytes.

FigS.3

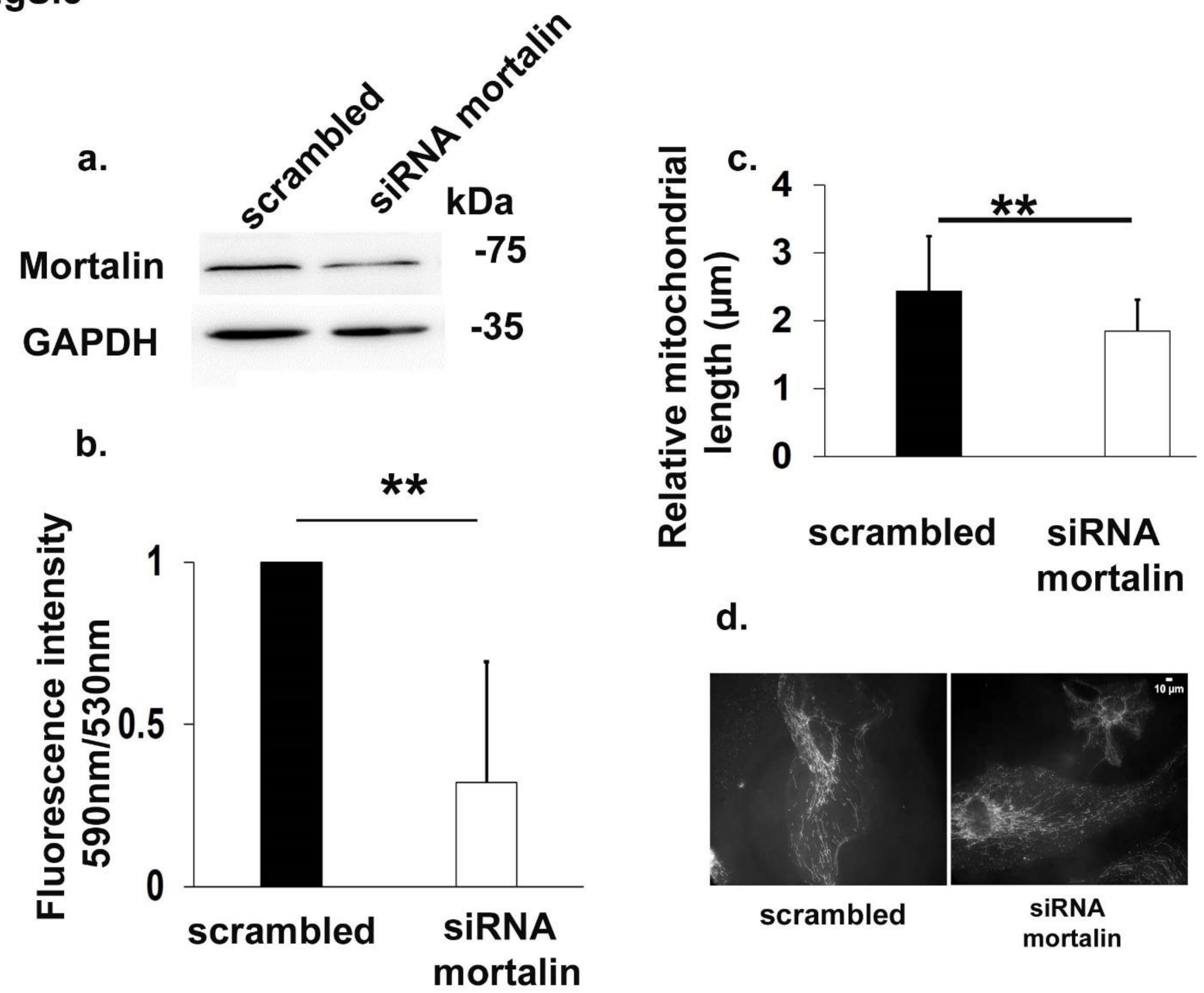

FigS.3) Knockdown of mortalin in PDA damages the mitochondrial health. a) western blot represents the knockdown of mortalin in PDA b) Fold change in mitochondrial membrane potential (mean fluorescence intensity of JC-1 aggregates/JC-1 monomers) in indicated transfected groups after 24h, as analyzed by fluorescence estimation (n=3). **c**) Quantitative analysis of mitochondrial length stained with mitotrackerRED (n=3). d) Representative images of PDA post 24h of transfection, stained with mitotrackerRED, Scale bar 10μm (n=3). All data represent mean ± standard deviation, from independent experiments (n stands for number of independent experiment,**p<0.005 with respect to control.

FigS.4

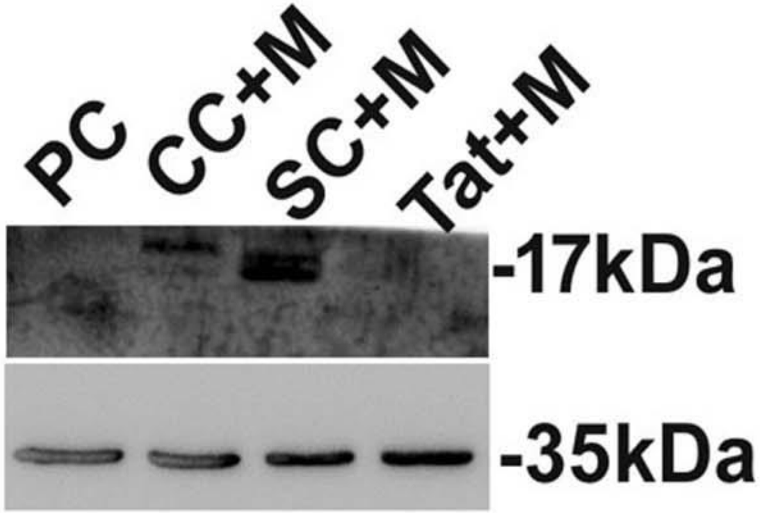

FigS.4) Expression of mutant Tat. Representative western blot shows the expression of HIV-1 Tat in cotransfected PDA. Abbreviations are as follows: CC (cysteine-cysteine) and SC (serine-cysteine) are the isogenic mutant of wild type Tat-C, M-Mortalin and Tat- HIV-1 Tat.

FigS.5

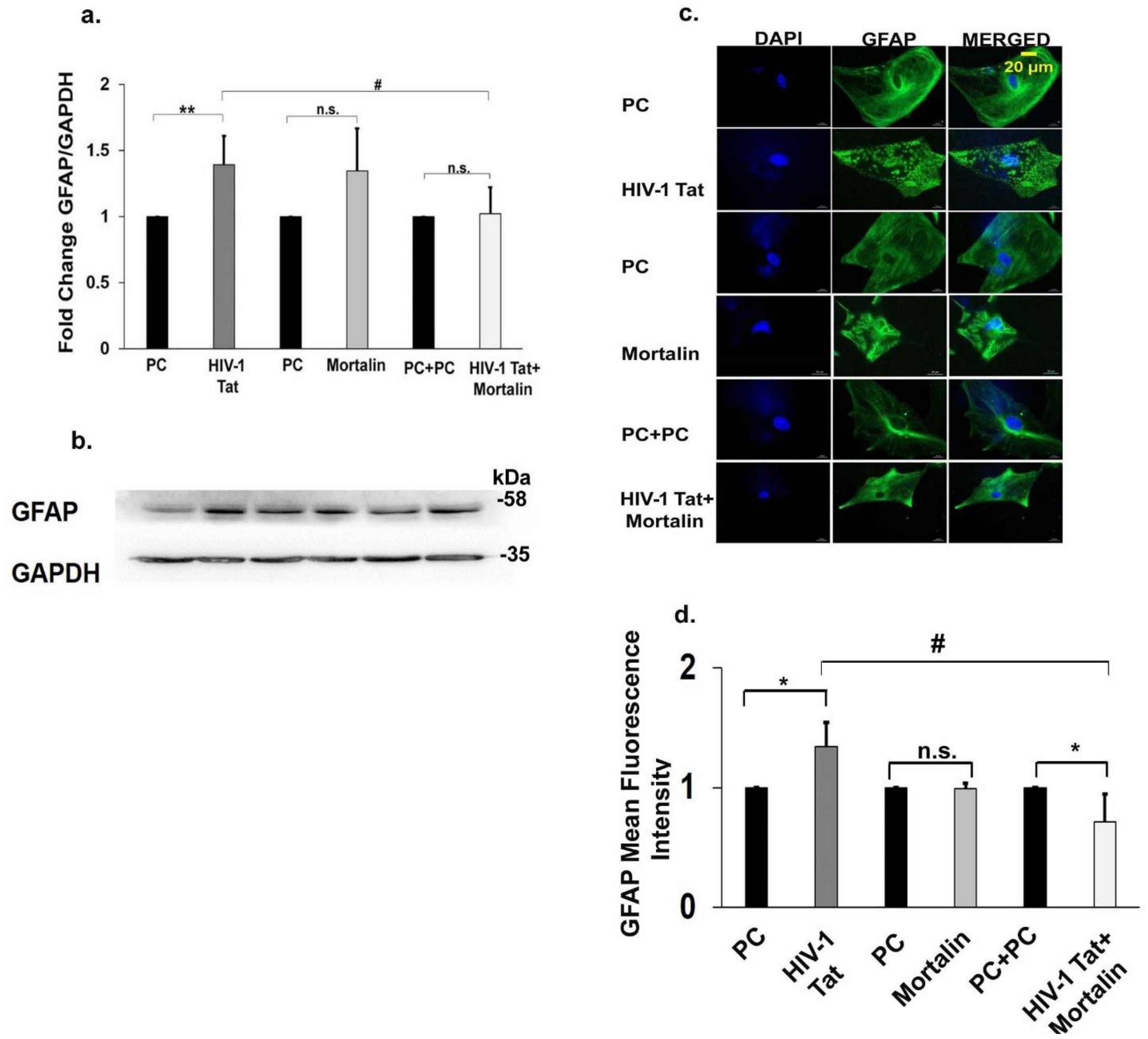

FigS.5) GFAP Expression and Aggregation in progenitor derived astrocytes. a) Fold change in the expression of GFAP in transfected astrocytes (n=4). b) Representative western blot showing change in GFAP at protein level (n=4). c) Immunocytochemistry of GFAP in green counterstained with DAPI, showing aggregates of intermediate filaments. Scale bar, 20µm. d) Fold change in the mean fluorescence intensity of GFAP (n=3). All data represent mean ± standard deviation, from independent experiments (n stands for number of independent experiment, *p<0.05, **p<0.005 with respect to control, $^{\#}$p<0.05, $^{\#\#}$p<0.005 with respect to HIV-1 Tat and Cotransfected group. (PC stands for Plasmid control)

FigS.6

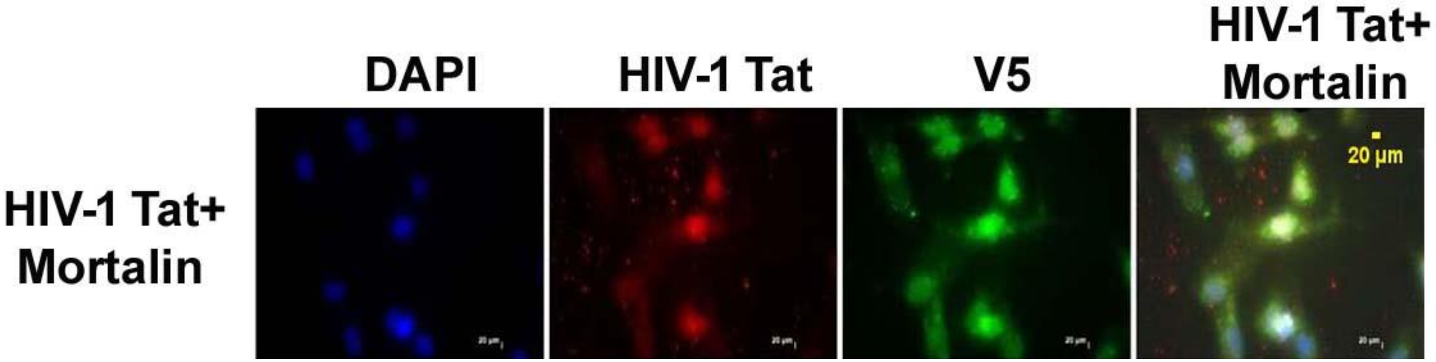

FigS.6. Expression of HIV-1 Tat in cotransfected PDA. Representative immunofluorescence image of cotransfected PDA showed the expression of Tat and exogenous mortalin tagged with V5 after 24h of transfection. Scale bar 20µm. (n=3)
